# Aptamer‐Engineered Liposomal Platform Enables in Situ cDC1 Vaccination to Potentiate Immunotherapy in Prostate Cancer

**DOI:** 10.1002/advs.202522169

**Published:** 2026-02-24

**Authors:** Jiayi Wang, Xuan Wang, Xinfeng Dai, Linxin Tian, Wencheng Shen, Xueliang Liu, Wei Xue, Jiahua Pan, Yu Yang

**Affiliations:** ^1^ Institute of Molecular Medicine (IMM) and Department of Urology Renji Hospital School of Medicine Shanghai Jiao Tong University Shanghai China; ^2^ Punan Branch of Renji Hospital Shanghai Jiaotong University School of Medicine Shanghai China; ^3^ School of Life Science Shanghai University Shanghai China; ^4^ College of Chemistry and Materials Science Shanghai Normal University Shanghai China

**Keywords:** aptamer, immune checkpoint blockade (ICB), in situ cDC1 vaccination, liposome, prostate cancer (PCa)

## Abstract

Prostate cancer (PCa) is a prototypical “cold tumor” with low immunogenicity and a highly immunosuppressive tumor microenvironment, which severely limits the efficacy of immunotherapy. Notably, the number and functionality of conventional type 1 dendritic cells (cDC1), which are critical antigen‐presenting cells, are markedly reduced in PCa, thereby impairing T cell priming. Here, we developed an aptamer‐modified liposomal platform (Apt‐Flt3L@Lipo) for the targeted co‐delivery of Flt3 ligand (Flt3L) and chlorin e6 (Ce6) to elicit in situ cDC1 vaccination. Upon ultrasound activation, Ce6 induced immunogenic cell death (ICD) in tumor cells, releasing abundant tumor antigens, while the concurrently released Flt3L promoted the differentiation and intratumoral recruitment of cDC1s. Thus, by coupling antigen release with cDC1 activation, we generated an in situ cDC1 vaccine, which enhanced antigen cross‐presentation, primed CD8^+^ T cells, and robustly suppressed tumor progression. When combined with immune checkpoint blockade (ICB), the strategy synergistically controlled both primary and distant tumors. In a patient‐derived ex vivo explant and transwell validation models, this approach demonstrated strong translational potential for inducing effective anti‐tumor immunity. Collectively, this platform offered a novel immunotherapy strategy for treating immunologically “cold” prostate cancer with strong potential for clinical translation.

## Graphical abstract

Prostate cancer is immunologically ‘cold’, with scarce, dysfunctional type 1 conventional dendritic cells (cDC1s) that limit T cell priming. We introduce an aptamer‐targeted liposomedelivering FMS‐like tyrosine kinase 3 ligand (Flt3L) and chlorin e6 (Ce6). Ultrasound induces antigen release and cDC1s recruitment, creating an in situ cDC1 vaccine. This reshapes the tumor microenvironment and activates CD8^+^ T cells, enhancing immune checkpoint blockade efficacy.

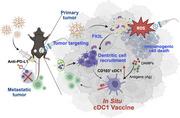

## 1 Introduction

Prostate cancer (PCa) is the most common malignancy of the male genitourinary system and the second leading cause of cancer‐related death among men in many developed countries [1]. While radical prostatectomy remains the preferred treatment for early‐stage PCa, approximately 30% of patients are diagnosed at an advanced stage or with distant metastases. This renders them ineligible for radical surgery or radiotherapy, resulting in a poor 5‐year survival rate of only ∼30% [2]. In recent years, immune checkpoint blockade (ICB) therapies have achieved remarkable success across various cancer types [1, 3, 4]. However, clinical responses in PCa have been limited, primarily due to its prototypical “cold tumor” characteristics‐namely, low tumor mutational burden, insufficient neoantigen generation, sparse infiltration of effector immune cells, and an immunosuppressive tumor microenvironment (TME) enriched with regulatory T cells (Tregs), myeloid‐derived suppressor cells (MDSCs), and inhibitory cytokines such as IL‐10 and VEGF [5, 6, 7, 8]. These factors collectively impair the initiation and maintenance of effective anti‐tumor immune responses [8, 9, 10, 11]. Therefore, strategies that can reshape the immune landscape to promote infiltration and activation of effector immune cells are urgently needed to enhance the immunotherapy efficacy in PCa.

Dendritic cells (DCs) are central regulators of antitumor immunity and hold significant clinical relevance [12, 13, 14]. Sipuleucel‐T, the only FDA‐approved therapeutic DC vaccine for PCa, works by ex vivo activating autologous DCs with a defined antigen. The engineered DCs are then reinfused into patients to stimulate T cell‐mediated immune responses [15, 16, 17]. However, this approach faces several limitations, including restricted antigen breadth, complex manufacturing procedures, potential functional impairment of DCs during ex vivo handling, and rapid functional decline upon reintroduction into the immunosuppressive TME [18, 19]. In contrast, endogenous in situ tumor vaccination (EITV) strategies aim to induce immunogenic cell death (ICD) of tumor cells in vivo [20, 21], thereby releasing a full repertoire of patient‐specific tumor antigens without the need for antigen selection or ex vivo manipulation [22], an approach that holds promise for overcoming existing vaccine limitations [23, 24, 25].

**FIGURE 1:**
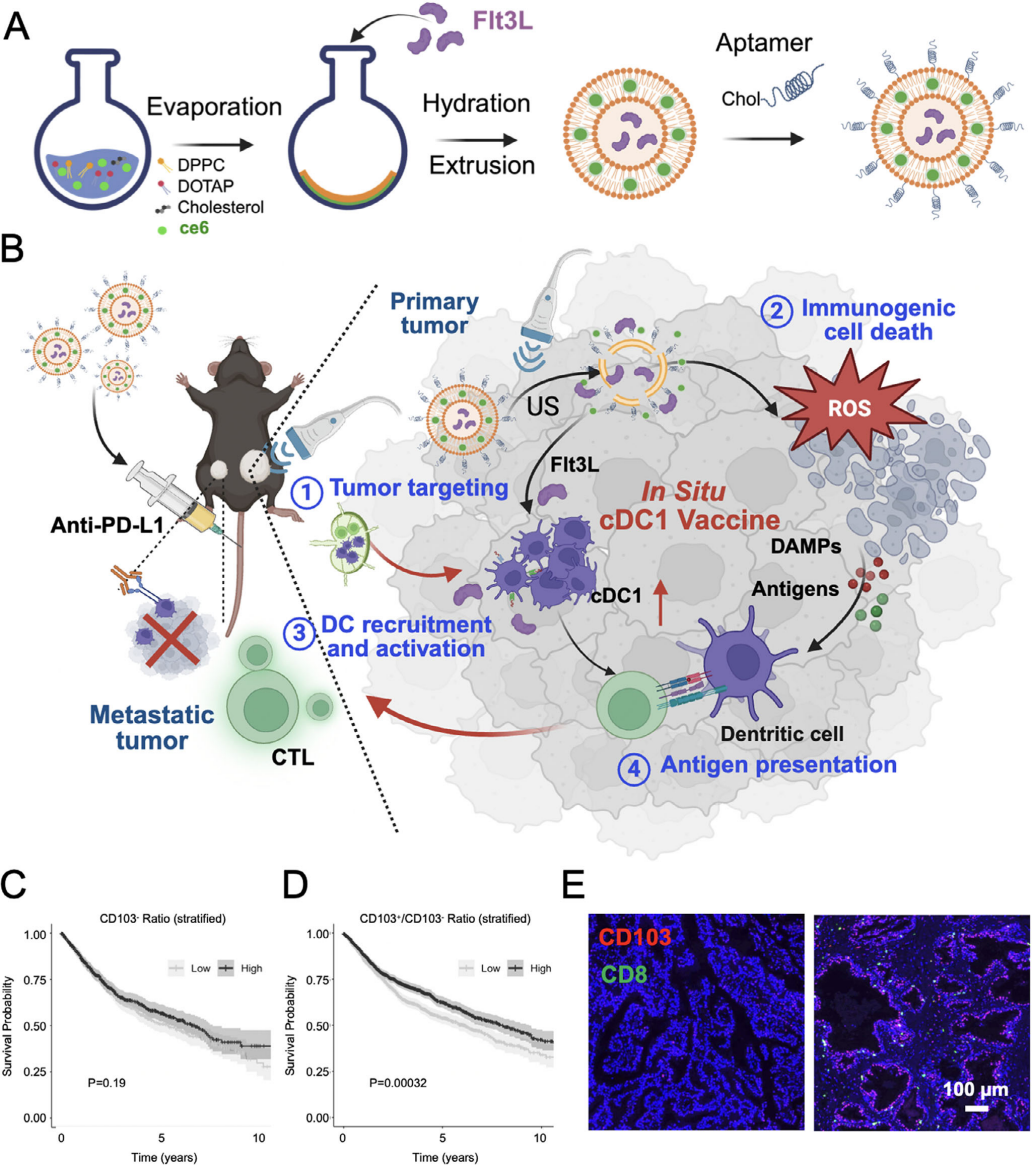
Schematic illustration showing (A) the fabrication of Apt‐Flt3L@Lipo and (B) its performance in inducing in situ cDC1 vaccine in prostate tumors. Apt‐Flt3L@Lipo was prepared by thin‐film hydration, extrusion, and EpCAM aptamer conjugation. Intravenous Apt‐Flt3L@Lipo selectively targeted prostate tumors via aptamer‐mediated delivery. Ultrasound‐activated Ce6 induced immunogenic cell death (ICD) and tumor antigen release. The concurrently released Flt3L recruited and activated the conventional type 1 dendritic cells (cDC1), enhancing antigen presentation and T cell priming. This combined strategy induced an in situ cDC1 vaccine, effectively inhibiting primary tumor growth and reducing metastatic tumor burden. (C) Kaplan–Meier survival analysis across all 10 cancer types in human TCGA data sets (PRAD, BLCA, BRCA, GBM, HNSC, KIRC, LAML, LUAD, LUSC, UCEC), adjusting for cancer type based on high CD103^−^ gene ratio. (D) Kaplan–Meier survival analysis across all 10 cancer types in human TCGA data sets, adjusting for cancer type based on the high CD103^+^/CD103^−^ gene ratio. (E) Representative immunofluorescence (IF) images of human prostate cancer samples showing CD103^+^ (red) and CD8^+^ (green) expression.

However, DCs subsets within the TME are functionally heterogeneous [12, 15, 26, 27]. While plasmacytoid DCs (pDCs) and tolerogenic DCs (tolDCs) often exhibit immunosuppressive profiles, conventional type 1 DCs (cDC1s) are uniquely capable of cross‐presenting antigens and orchestrating cytotoxic T cell responses, making them indispensable for effective anti‐tumor immunity [28, 29, 30]. cDC1s efficiently process and present tumor antigens via the MHC class I pathway, secrete chemokines such as CXCL9/10 to recruit effector T cells to the tumor site, and maintain T cell activation through IL‐12 production [31, 32, 33, 34]. Our bioinformatic analysis of The Cancer Genome Atlas (TCGA) revealed no significant correlation between overall DCs infiltration and patient overall survival (OS) in PCa (*p* >0.1, Figure 1C), whereas high infiltration of CD103^+^ cDC1s was strongly associated with improved OS (*p* <0.001, Figure 1D). Moreover, histopathological analysis of patient‐derived PCa specimens showed significantly higher CD8^+^ T cell infiltration in CD103^+^ regions compared to CD103^−^ regions (Figure 1E). These findings underscore the central role of cDC1s in shaping productive T cell immunity and provide a compelling rationale for therapeutic strategies targeting cDC1s to boost anti‐tumor responses.

Here, we propose an “in situ cDC1 vaccine” strategy, designed to selectively activate cDC1s within the tumor milieu and induce in situ uptake and presentation of autologous tumor antigens, thereby generating potent, antigen‐specific T cell responses without the need for ex vivo manipulation. To this end, we engineered an aptamer‐modified liposomal delivery platform (Apt‐Flt3L@Lipo) for the targeted co‐delivery of chlorin e6 (Ce6) and Fms‐like tyrosine kinase 3 ligand (Flt3L). Upon intravenous administration, Apt‐Flt3L@Lipo preferentially accumulated in PCa tissues. After ultrasound activation, Ce6 induces ICD to release tumor antigens, while Flt3L promotes cDC1 recruitment and maturation. This dual‐action strategy establishes an in situ cDC1 vaccine, wherein activated cDC1s capture and cross‐present autologous antigens to CD8^+^ T cells, thereby initiating a potent tumor‐specific immune response and reversing the immunosuppressive microenvironment of “cold” PCa.

Furthermore, this strategy upregulates PD‐L1 expression within the cDC1 cells, thereby enhancing synergy with ICB therapy. In patient‐derived PCa models, Apt‐Flt3L@Lipo significantly enhanced responsiveness to immunotherapy, demonstrating strong potential for clinical translation in the treatment of low‐immunogenicity PCa.

## 2 Results

### 2.1 Preparation and Characterization of Apt‐Flt3L@Lipo

**FIGURE 2:**
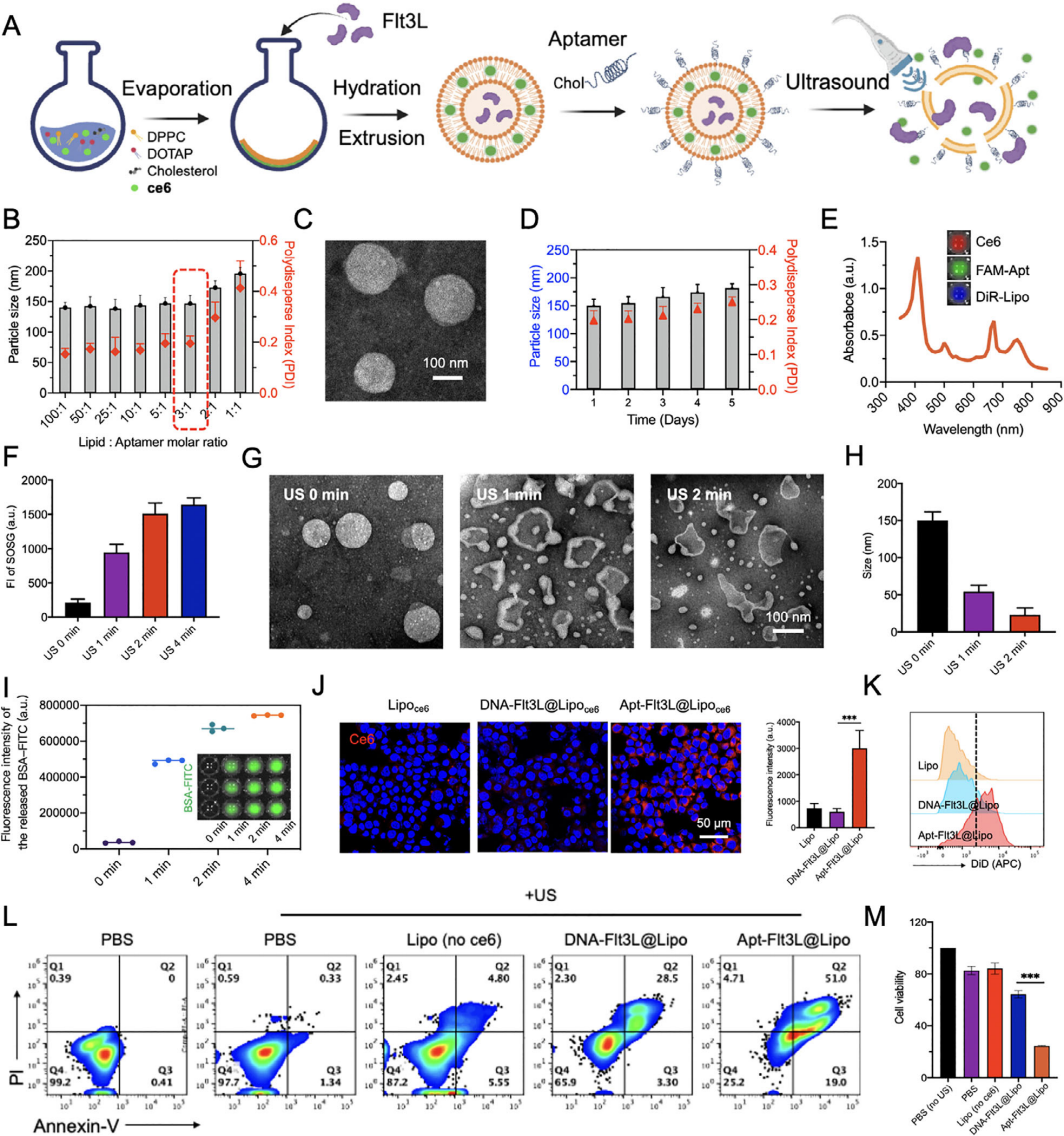
Characterization and functional validation of Apt‐Flt3L@Lipo. (A) Schematic representation of the Apt‐Flt3L@Lipo formulation process, including evaporation, hydration, extrusion, and aptamer modification steps. (B) Particle size and polydispersity index (PDI) of Apt‐Flt3L@Lipo synthesized at different lipid‐to‐aptamer molar ratios. Data showed an optimal size and PDI at a specific ratio (highlighted in red). (C) Transmission electron microscopy (TEM) image of Apt‐Flt3L@Lipo at a 1:3 lipid‐to‐aptamer molar ratio showing a uniform spherical morphology with a diameter of approximately 100 nm. (D) Stability assessment of Apt‐Flt3L@Lipo in PBS at 4 °C over 5 days, with measurements of particle size and PDI indicating structural integrity. (E) Absorbance spectra of Apt‐Flt3L@Lipo, displaying characteristic peaks of Ce6, FAM‐labeled aptamer, and DiR‐labeled liposome, confirming the presence of each component. Fluorescence imaging was conducted using DiR‐labeled liposome, FAM‐labeled aptamer, and Ce6 in a 96‐well plate with the IVIS Spectrum CT system, further verifying component incorporation. (F) SOSG fluorescence indicating ROS generation after different US treatment. (G) TEM images of Apt‐Flt3L@Lipo before (0 min) and after (1 and 2 min) US treatment, illustrating structural disruption over time. (H) DLS analysis of particle size reduction following US treatment, showing a size decrease correlated with US exposure. (I) US‐guided release of FITC‐labeled BSA from Apt‐BSA@Lipo, which were synthesized by the same method with Apt‐Flt3L@Lipo. The released BSA‐FITC was also visualized by the IVIS Spectrum CT system. (J) Confocal microscopy and fluorescence intensity quantification of intracellular Ce6 accumulation in RM1 prostate cancer cells treated with Lipo_ce6_, DNA‐Flt3L@Lipo_ce6_, and Apt‐Flt3L@Lipo_ce6_. (K) Flow cytometry analysis of Ce6 fluorescence in RM1 cells after incubation with Lipo_ce6_, DNA‐Flt3L@Lipo_ce6_, and Apt‐Flt3L@Lipo_ce6_. (L) Cell apoptosis induced by Apt‐Flt3L@Lipo was examined via flow cytometry. The cells were stained with Annexin V‐FITC and PI. (M) Cell viability assay (CCK‐8) of RM1 cells treated with Apt‐Flt3L@Lipo under US. Data are presented as mean ± s.d. ^*^ *p* <0.05, ^**^ *p* <0.01, and ^***^ *p* <0.001.

The stepwise preparation of the Aptamer‐Engineered Liposomal Platform (Apt‐Flt3L@Lipo) involved thin‐film hydration, extrusion, and subsequent conjugation with EpCAM aptamer [35, 36] (Figure 2A). Specifically, hydrophobic Ce6 was added into the phospholipid‐containing organic phase (DPPC, DOTAP, and cholesterol) for coencapsulation. After evaporation, the hydration process was then carried out with deionized water or PBS containing Flt3L, and the electrostatic interaction of the cationic DOTAP enabled efficient Flt3L encapsulation [37]. After filtration through 400, 200, and 100 nm filters using Avanti miniextruder, cholesterol‐conjugated EpCAM aptamers were added, anchoring the aptamer to the liposome surface via the hydrophobicity of cholesterol to generate the aptamer‐engineered liposomal platform (Apt‐Flt3L@Lipo).

To optimize the aptamer‐engineered liposomal formulation, we tested various lipid‐to‐aptamer molar ratios and evaluated their effects on particle size and polydispersity index (PDI) (Figure 2B). Through a comprehensive evaluation of liposome‐to‐aptamer ratios ranging from 100:1 to 1:1, we found that at a liposome‐to‐aptamer ratio of 3:1 (molar lipid: molar aptamer), dynamic light scattering (DLS) revealed an average liposome size of 146.8 nm and a PDI of 0.195, indicating optimal stability. Transmission electron microscopy (TEM) images (Figure 2C) confirmed the spherical morphology of the Apt‐Flt3L@Lipo with an average diameter of around 100 nm, aligning with DLS measurements. The stability of Apt@Lipo was evaluated over five days (Figure 2D). The results indicated that both particle size and PDI remained consistent, suggesting that the liposomes maintain structural integrity under physiological conditions. Overall, these results demonstrated that a 3:1 liposome‐to‐aptamer ratio offered optimal conditions for generating stable and uniform aptamer‐engineered liposomes, making them suitable for subsequent biological applications.

The successful functionalization of liposomes with Flt3L and aptamer was also confirmed by measuring the particle size and zeta potential (Figure S1). The results showed that the size of the liposomes remained largely unchanged after Flt3L and aptamer conjugation, indicating that functionalization did not significantly alter the structural integrity of the vesicles. Zeta potential measurements showed that pristine liposomes (Lipo) exhibited a relatively high positive zeta potential due to the presence of cationic lipids. Upon conjugation with Flt3L, a slight reduction in zeta potential was observed, suggesting successful incorporation of the cytokine. The most pronounced charge reduction occurred after aptamer conjugation, confirming successful surface modification of the aptamer. To further characterize the Apt‐Flt3L@Lipo formulation, the aptamer and liposomes were labeled with FAM and DiR, respectively. The resulting triple‐labeled liposome formulation (DiR, FAM, and ce6) exhibited four distinct peaks in the absorbance spectrum: peaks at 410 and 690 nm were attributed to ce6, 488 nm to FAM, and 750 nm to DiR. The fluorescence from the triple‐labeled liposome formulation was then visualized using an IVIS spectrum imaging system in a 96‐well plate (Figure 2E). In conclusion, the successful co‐encapsulation of Flt3L and Ce6, combined with surface modification of EpCAM aptamer, demonstrated the structural integrity and functional efficacy of this liposomal formulation, supporting its potential for further biological applications.

Furthermore, a singlet oxygen green probe (SOSG) was used to investigate the sonodynamic efficiency of Apt‐Flt3L@Lipo under US mediation [38]. The results showed that, compared to the untreated control, the fluorescence intensity of the SOSG increased by 4.36, 6.96, and 7.58 times after 1, 2, and 4 min of US exposure, respectively (Figure 2F), indicating the generation of ce6‐mediated ROS. In addition, we further investigated the US‐mediated controlled drug release performance of the Apt‐Flt3L@Lipo. TEM results showed that at 0 min, the liposome exhibited well‐defined, spherical morphologies, while significant structural collapse and fragmentation were observed after 1 and 2 min of US exposure, indicating their sensitivity to mechanical disruption by US (Figure 2G). Consistent with these morphological changes, DLS analysis showed a marked reduction of particle size with increasing US exposure time, confirming the breakdown of the liposomal structure into smaller fragments (Figure 2H). To further validate the US‐responsive protein release properties, FITC‐labeled BSA was encapsulated in Apt‐BSA@Lipo, a liposome analog synthesized using the same method as Apt‐Flt3L@Lipo. Upon US treatment, a time‐dependent release of FITC‐BSA was observed, which was concurrently monitored using the IVIS Spectrum CT system (Figure 2I). Quantitative analysis revealed a direct correlation between US exposure duration and protein release efficiency, confirming the precisely controllable drug release profile of this system. Additionally, we assessed the cytotoxicity of Apt‐Flt3L@Lipo at different Ce6 concentrations. At a ce6 concentration of 0.5 µm, cell viability remained above 95%, demonstrating the biocompatibility of this system (Figure S2). This concentration was selected as the therapeutic dose for subsequent experiments.

### 2.2 EpCAM Aptamer‐Engineered Liposomes Achieve Tumor‐Targeted Enhancement of SDT In Vitro

Epithelial cell adhesion molecule (EpCAM) is a transmembrane glycoprotein highly expressed in proliferative cancers, with previous studies reporting consistent and elevated expression of EpCAM in both primary and metastatic PCa tissues [39, 40]. To investigate the enhanced targeting capability of EpCAM aptamer‐engineered liposomes toward PCa in vitro, unmodified Lipo_ce6_ and random aptamer‐modified DNA‐Flt3L@Lipo were used as controls. In vitro, confocal microscopy was employed to analyze Ce6 fluorescence after incubating Apt‐Flt3L@Lipo with RM1 prostate cancer cells for 0.5, 1, and 2 h. The results showed a time‐dependent increase of Ce6 fluorescence in Apt‐Flt3L@Lipo group (Figure 2J; Figure S3), with Ce6 fluorescence intensity at 2 h being 4.07‐fold and 5.01‐fold higher than that of Lipo_ce6_ and DNA‐Flt3L@Lipo_ce6_, respectively, demonstrating the efficient PCa cell‐targeting effect mediated by EpCAM aptamer modification. Additionally, flow cytometry analysis further confirmed that the number of ce6‐positive cells in the Apt‐Flt3L@Lipo group was significantly higher than in the control groups (Figure 2K), further demonstrating the high‐efficiency tumor‐targeting capability mediated by the EpCAM aptamer‐engineered liposomes.

Upon successfully synthesizing and verifying the targeting capabilities of Apt‐Flt3L@Lipo, we proceeded to investigate its tumor‐targeted SDT effects. RM1 cells were initially incubated with Apt‐Flt3L@Lipo, followed by washing and exposure to US (10 W, 50%) for 2 min. Firstly, to assess ROS generation under US treatment, DCFH probe imaging was used after US treatment. The results showed that DCFH fluorescence in the Apt@Lipo group was 5.87 times and 3.02 times higher than in the Lipo and Lipo_ce6_ groups, respectively (Figure S4), confirming enhanced ROS production after US medicated by EpCAM modification. Cell apoptosis was then evaluated using Annexin V/Propidium Iodide staining. The results revealed that Ce6‐loaded Lipo_ce6_ (Flt3L@Lipo_ce6_) significantly induced apoptosis, validating Ce6's effectiveness as a sonosensitizer for US. Moreover, Apt‐Flt3L@Lipo elicited even more apoptosis than Lipo_ce6_, underscoring the enhanced tumor‐killing efficiency conferred by EpCAM aptamer‐mediated tumor targeting (Figure 2L). A CCK‐8 assay further demonstrated that the cell viability in the Apt‐Flt3L@Lipo group was 24.4%, significantly lower than that in the Lipo and Lipo_ce6_ control groups (Figure 2M), highlighting the enhanced tumor cell‐killing ability of US through targeted delivery. These results indicated that aptamer engineering significantly enhances the US‐mediated tumor‐killing efficiency of Apt‐Flt3L@Lipo by promoting targeted delivery and increased ROS production.

### 2.3 Apt‐Flt3L@Lipo Combined With SDT Achieved In Situ cDC1 Vaccine and Robust Anti‐Tumor Immune Activation

**FIGURE 3:**
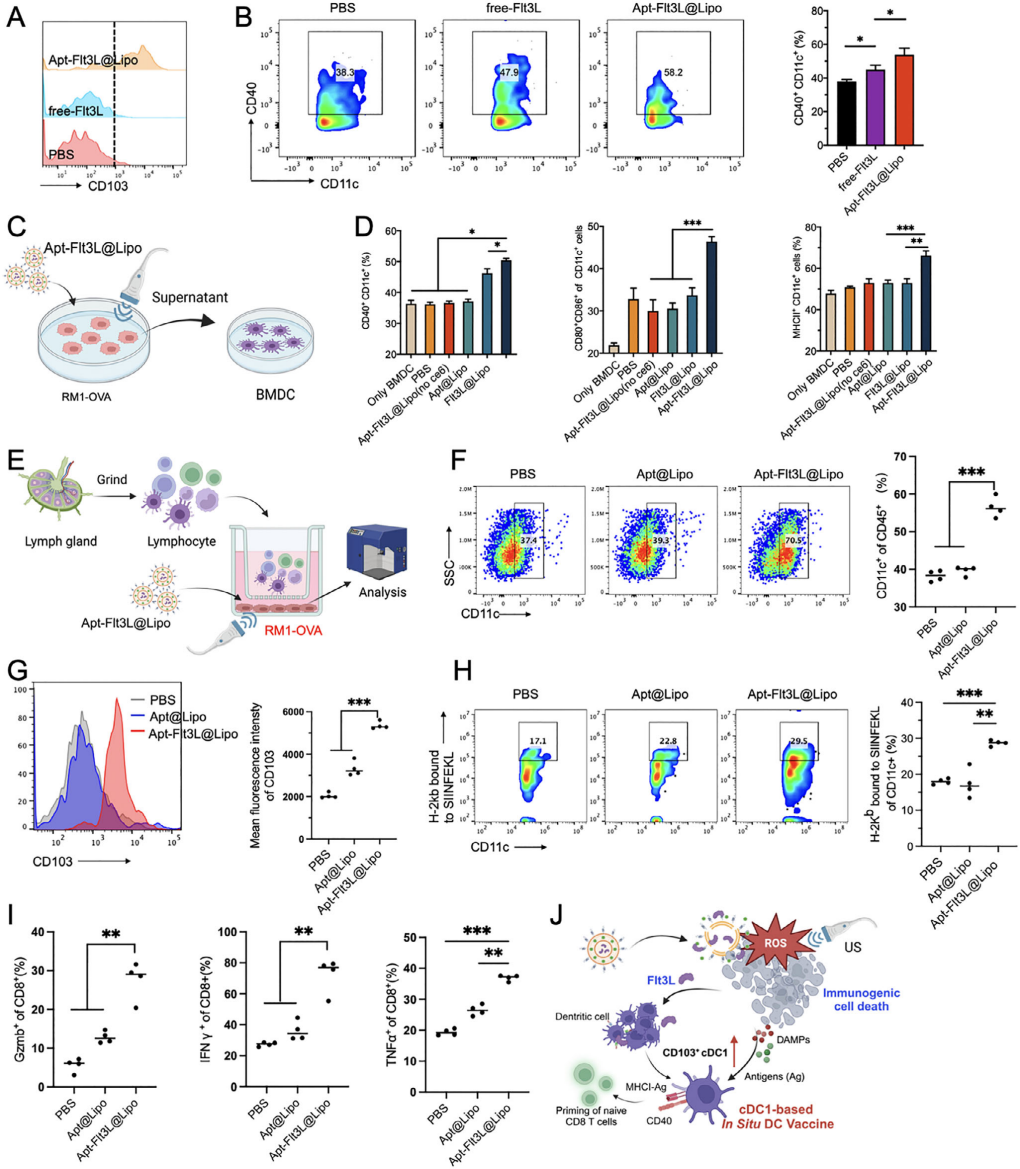
Targeted co‐delivery of Flt3L/Ce6 by Apt‐Flt3L@Lipo enabled cDC1‐based vaccination in vitro. (A,B) Flow cytometry of bone marrow‐derived dendritic cells (BMDCs) showing activation markers CD40^+^ and differentiation markers CD103^+^ after treatment with PBS, free‐Flt3L, or Apt‐Flt3L@Lipo. (C) Schematic diagram illustrating the experimental design for co‐culturing BMDCs with the supernatant from RM1‐OVA cells treated with Apt‐Flt3L@Lipo + US. (D) Flow cytometry analysis of BMDCs co‐incubated with the supernatant from treated RM1‐OVA cells showed significantly increased expression of activation markers (CD40^+^, CD80^+^CD86^+^) and antigen‐presentation marker (MHCII^+^) among CD11c^+^ cells, confirming enhanced immune stimulation by Apt‐Flt3L@Lipo + US. (E) Transwell migration assay design illustrating the recruitment of lymph node‐derived immune cells toward RM1‐OVA cells treated with Apt‐Flt3L@Lipo under US. (F) Flow cytometry analysis revealed increased migration of CD11c^+^ DCs in response to Apt‐Flt3L@Lipo + US‐treated RM1 cells, indicating effective immune cell recruitment. (G) Differentiation of CD103^+^ cDC1 was significantly enhanced by Apt‐Flt3L@Lipo + US, as shown by flow cytometry, emphasizing its role in promoting the development of antigen‐presenting dendritic cell subsets. (H) Flow cytometry analysis of H‐2K^b^/SIINFEKL complexes on CD11c^+^ DCs showing a significant increase in antigen presentation in the Apt‐Flt3L@Lipo + US treatment group compared to PBS, indicating enhanced antigen cross‐presentation capability of DCs. (I) Flow cytometry analysis of IFN‐γ^+^, TNF‐α^+,^ and Gzmb^+^ of CD8^+^ T cells in the lymph node‐derived immune cells. (J) Schematic representation of the in situ cDC1 vaccine: The aptamer‐enginnered liposomal delivery of Ce6 induced reactive oxygen species (ROS)‐mediated immunogenic cell death, releasing tumor antigens, while Flt3L delivery promoted the recruitment and activation of CD103^+^ cDC1 cells in tumor microenvironment. The cDC1 cells phagocytose and present tumor antigens in situ, forming a potent in situ cDC1 vaccine, which activated T cells to kill tumor cells. Data are presented as mean ± s.d. ^*^ *p* <0.05, ^**^ *p* <0.01, and ^***^ *p* <0.001.

cDC1 are a specialized DCs subset distinguished by high MHC, CD40 expression, and unique cross‐presentation capacity, crucial for anti‐tumor immunity [41]. These cells are essential for antigen presentation and play a critical role in the efficacy of DC‐based vaccines [42]. Flt3L, through its interaction with the Flt3 receptor, supports the growth and differentiation of cDC1s, ensuring their functional activity [43]. In this work, we utilized an aptamer‐engineered liposomal platform to achieve targeted delivery of Flt3L to PCa, with the potential to significantly enhance the recruitment and differentiation of cDC1s within the tumor microenvironment. To test this, bone marrow‐derived dendritic cells (BMDCs) from male C57 mice were incubated with either free Flt3L or Apt‐Flt3L@Lipo for 2 h after US treatment for 2 min, DCs activation was then assessed by flow cytometry. The analysis showed an increase in CD11c^+^CD40^+^, CD11c^+^MHCII^+^, CD80^+^CD86^+^, and CD103^+^ cells after free Flt3L treatment (Figure 3A,B, Figures S5 and S6), indicating BMDC differentiation into functional DCs, particularly the cDC1 subset. Apt‐Flt3L@Lipo induced stronger activation and differentiation of cDC1 subset than free Flt3L, likely due to enhanced stability provided by the liposomal system, emphasizing the benefits of Flt3L delivery. To further evaluate whether Apt‐Flt3L@Lipo could mediate DCs activation through sonodynamic therapy (SDT), RM1‐OVA cells (ovalbumin‐expressing RM1 cell line) were treated with Apt‐Flt3L@Lipo for 2 h followed by ultrasound irradiation (10 W, 50% duty cycle, 2 min) (Figure 3C). The resulting supernatant, containing both tumor‐derived OVA antigen (released from RM1‐OVA cells) and Flt3L (released from Apt‐Flt3L@Lipo), was then incubated with BMDCs for 2 h. Flow cytometry analysis demonstrated that this treatment significantly upregulated the expression of MHCII^+^, CD40^+^, and CD80^+^CD86^+^ in CD11c^+^ cells (Figure 3D; Figure S7), suggesting effective differentiation and activation of BMDCs toward a cDC1 phenotype.

In addition, previous research has shown that Flt3L not only promotes cDC1 subset activation but also recruits DCs from tumor‐draining lymph nodes into the immunosuppressive tumor microenvironment [4]. To evaluate the DCs recruitment ability of Apt‐Flt3L@Lipo, we conducted a transwell migration assay (Figure 3E). RM1‐OVA cells treated with Apt‐Flt3L@Lipo and US were placed in the lower chamber, while single‐cell suspensions from mouse lymph nodes were placed in the upper chamber. Flow cytometry confirmed that CD11c^+^ DCs in the lower chamber increased by 27% and 25% after Apt‐Flt3L@Lipo treatment compared to PBS and Apt@Lipo (Figure 3F), with the CD103^+^ cDC1 subset increasing by 2.05 times, indicating that Apt‐Flt3L@Lipo effectively promotes DCs infiltration into tumors through the delivery of Flt3L (Figure 3G). Furthermore, a marked upregulation of H‐2K^b^/SIINFEKL complexes was observed in Apt‐Flt3L@Lipo‐treated CD11c^+^ dendritic cells, confirming enhanced antigen cross‐presentation capability (Figure 3H). Taken together, these findings suggest that the Flt3L component in Apt‐Flt3L@Lipo‐mediated SDT activated cDC1s, equipping them with functional properties akin to an in situ cDC1 vaccine, namely, mature, tumor antigen‐presenting cDC1 capable of priming antitumor immunity. Additionally, the infiltration of CD8^+^ T cells in tumor tissues significantly increased, accompanied by enhanced production of effector cytokines (IFN‐γ, TNF‐α), and release of cytolytic granules (Gzmb) (Figure 3I; Figure S8). This demonstrated that the SDT treatment mediated by Apt‐Flt3L@Lipo effectively activated T cell‐mediated tumor‐specific cytotoxicity by in situ inducing the cDC1 subset and promoting tumor antigen presentation. In summary, these findings demonstrated that Apt‐Flt3L@Lipo efficiently recruited and activated cDC1 while enhancing the cross‐presentation of ultrasound‐released tumor antigens, thereby functioning as an in situ cDC1 vaccine to elicit potent antigen‐specific immune responses (Figure 3J).

### 2.4 Apt‐Flt3L@Lipo Enhanced Tumor Targeting and Suppressed Tumor Growth in a Prostate Cancer Mouse Model

**FIGURE 4:**
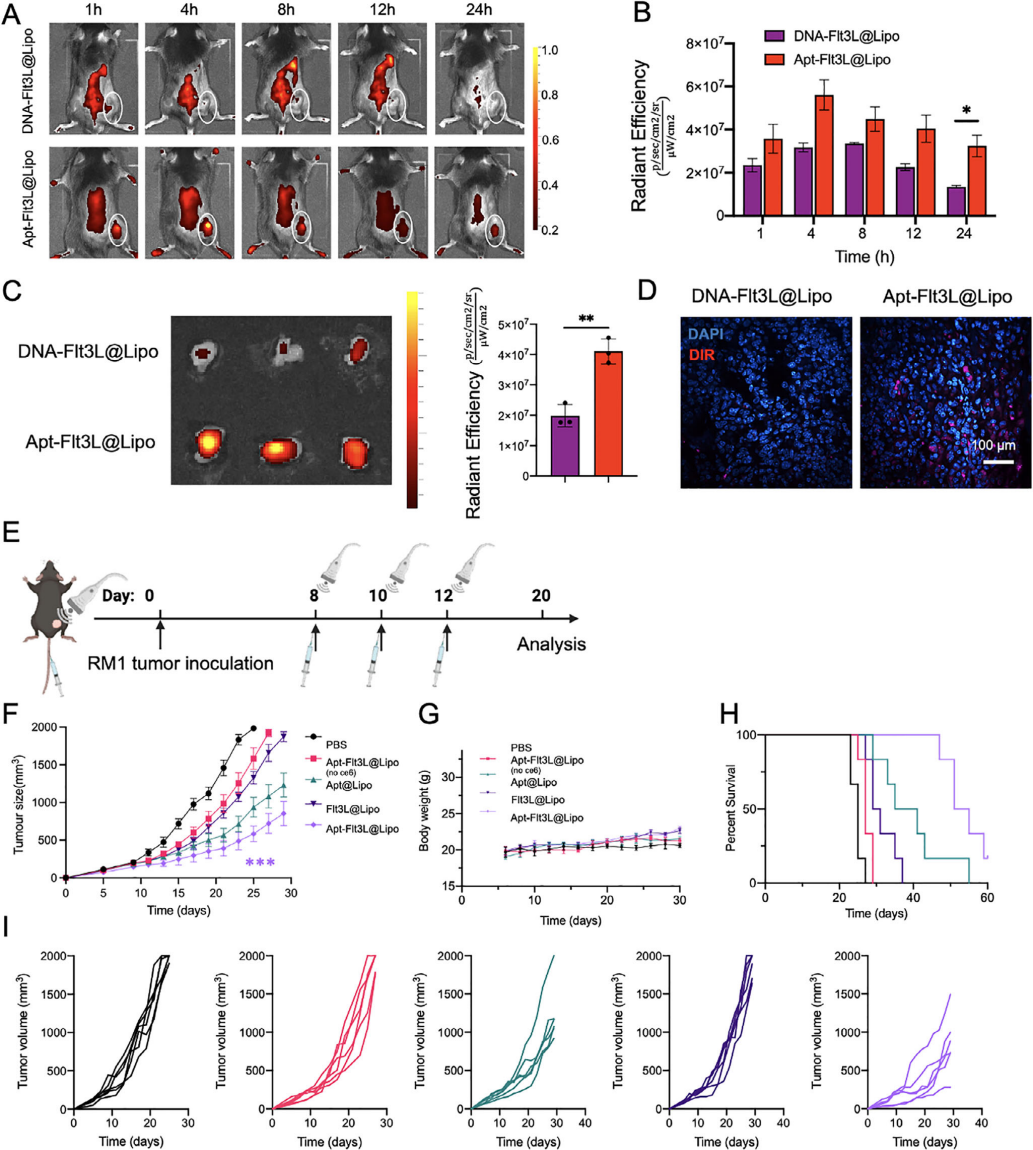
Apt‐Flt3L@Lipo targeted and suppressed prostate cancer in vivo. (A) Representative in vivo images and of (B) quantitative analysis tumor accumulation of DiR‐labeled DNA‐Flt3L@Lipo and Apt‐Flt3L@Lipo in RM1 tumor‐bearing mice at the indicated time points post‐injection (1, 4, 8, 12, and 24 h). (C) Ex vivo imaging of excised tumors after 24 h post‐injection of DiR‐labeled DNA‐Flt3L@Lipo and Apt‐Flt3L@Lipo and quantification analysis. (D) Micro‐distribution of DiR‐labeled DNA‐Flt3L@Lipo and Apt‐Flt3L@Lipo (Red) in tumors tissue sections. (E) Schematic representation of the in vivo experimental design using mice RM1 tumor models. (F) Comparison of the tumor volume across different treatment groups. (G) Mice body weight across different treatment groups. (H) Survival analysis of mice after different treatments, survival data are analyzed using Kaplan–Meier Method. (I) The individual tumor growth curves of RM1 tumor‐bearing mice that received various treatments. Data are presented as mean ± s.d. ^*^ *p* <0.05, ^**^ *p* <0.01, ^***^ *p* <0.001.

Before proceeding with in vivo treatment, we further investigated the tumor‐targeting capability of the EpCAM aptamer‐engineered Apt‐Flt3L@Lipo in vivo by establishing an RM1 prostate cancer tumor model. When the tumor volume reached 200 mm^3^, DiR‐labeled Apt‐Flt3L@Lipo and DNA‐Flt3L@Lipo were administered via intravenous injection, and DiR fluorescence intensity was monitored at 1, 4, 8, 12, and 24 h using the IVIS Spectrum CT imaging system. To quantitatively assess tumor accumulation, tumor regions of interest (ROIs) were manually delineated (white dashed circles in Figure 4A), and mean fluorescence intensity was calculated for each time point (Figure 4B). The results showed that DiR fluorescence intensity in the Apt‐Flt3L@Lipo group was significantly higher than that in the DNA‐Flt3L@Lipo group, demonstrating the superior in vivo targeting capability conferred by EpCAM aptamer modification. Notably, tumor‐associated fluorescence peaked at approximately 4 h and gradually declined thereafter, resulting in a higher signal at 12 h compared with 24 h. This pattern reflects the dynamic balance between tumor accumulation and systemic clearance of Apt‐Flt3L@Lipo. Based on this kinetic profile, 4 h post‐injection, corresponding to maximal tumor accumulation, was selected as the optimal time point for subsequent ultrasound treatment. After 24 h, the mice were sacrificed, and tumors were excised to further investigate the distribution of Apt‐Flt3L@Lipo in tumor tissue. The results indicated that accumulation in tumors from the Apt‐Flt3L@Lipo group was significantly higher than in the DNA‐Flt3L@Lipo group (Figure 4C), with fluorescence intensity 2.2‐fold higher, confirming the efficient tumor‐targeting effect. Additionally, immunofluorescence of tumor tissue sections showed that fluorescence in the Apt‐Flt3L@Lipo group was significantly higher than in the Lipo_ce6_‐Apt_Lib_ group (Figure 4D). Taken together, these in vivo and ex vitro experiments demonstrated that the EpCAM aptamer engineering significantly enhanced the tumor‐targeting ability of Apt‐Flt3L@Lipo toward PCa.

After confirming the tumor‐targeting capability of Apt‐Flt3L@Lipo in vivo, we further evaluated its in vivo treatment efficacy using a mouse PCa model, as illustrated in Figure 4E. On day 0, 5 × 10^6^ RM1 prostate cancer cells were injected into the right dorsal region of the mice. On day 8, the primary tumor volume reached approximately 150 mm^3^, and mice were randomly assigned to five treatment groups: PBS, Apt‐Flt3L@Lipo (no ce6), Flt3L@Lipo, Apt@Lipo, and Apt‐Flt3L@Lipo. All liposome drugs were dissolved in 100 µL saline prior to intravenous injection. Four h post‐injection, tumors were subjected to US at 10 W, 50% for 2 min. Treatments were repeated every two days for a total of three times. Tumor growth, body weight, and survival rates were monitored. The results showed that tumor growth in the Apt‐Flt3L@Lipo group was significantly suppressed compared to other groups, with the majority of mice (4/8) having tumor volumes below 1000 mm^3^ at day 29 (Figure 4F,I). These results indicated that the therapeutic effect of Apt‐Flt3L@Lipo was superior to Apt@Lipo alone, demonstrating that the addition of Flt3L further enhanced tumor control when combined with US. Body weight changes were similar across all groups during the treatment period (Figure 4G). Moreover, tumor suppression in the Apt‐Flt3L@Lipo group led to prolonged survival, 33.3% of mice (2/6) survived for 60 days, while only two mice (2/24) in all other groups survived beyond 40 days (Figure 4H).

### 2.5 Apt‐Flt3L@Lipo Reversed the Immunosuppressive Tumor Microenvironment and Enhanced Immune Checkpoint Blockade Therapy

**FIGURE 5:**
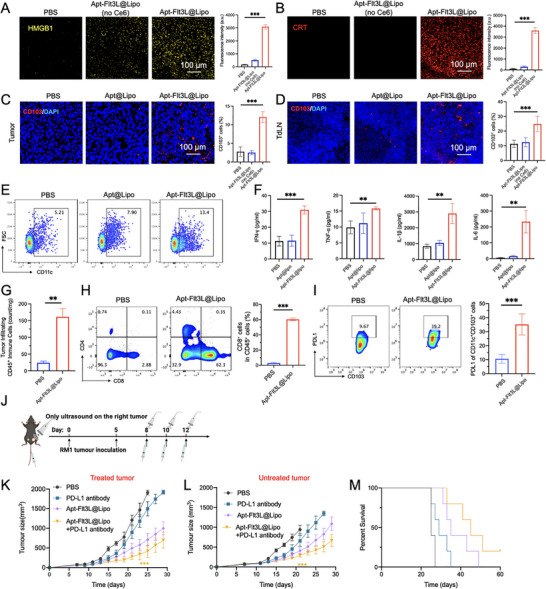
Apt‐Flt3L@Lipo reversed the immunosuppressive tumor microenvironment and enhanced immune checkpoint blockade therapy in a murine prostate cancer model. (A) Representative immunofluorescence images and quantification of (A) HMGB1 and (B) calreticulin (CRT) expression in tumor tissues from PBS‐ and Apt‐Flt3L@Lipo‐treated mice (scale bars: 100 µm). Immunofluorescence staining for CD103^+^ DCs (red) in (C) tumor tissues and (D) tumor‐draining lymph nodes (TdLNs) with corresponding quantification. (E) Flow cytometric analysis of intratumoral DCs showing elevated CD11c^+^ populations upon Apt‐Flt3L@Lipo administration. (F) Cytokine profiling of tumor lysates revealed significantly increased levels of IFN‐γ, IL‐1β, IL‐6, and TNF‐α in the Apt‐Flt3L@Lipo group compared to PBS controls. (G) Absolute numbers of tumor‐infiltrating CD45^+^ immune cells per milligram of tumor tissue in PBS‐ and Apt‐Flt3L@Lipo–treated mice. (H) Representative flow cytometry plots showing CD4 and CD8 expression among tumor‐infiltrating CD45^+^ immune cells, along with quantitative analysis of the percentage of CD8^+^ T cells within the CD45^+^ population. (I) Flow cytometry analysis of PD‐L1 expression on CD103^+^ dendritic cells from tumor samples, with quantified percentages. (J) Schematic of distant tumor model establishment and anti‐PD‐L1 antibody treatment timeline. (K) Tumor growth curves of right‐side (treated) tumors in mice receiving different treatment regimens (PBS, PD‐L1 antibody, Apt‐Flt3L@Lipo, or combination). (L) Tumor growth curves of left‐sided (untreated) tumors under the same treatment conditions. (M) Kaplan–Meier survival curves of tumor‐bearing mice in each treatment group. Data are presented as mean ± s.d. ^*^ *p* <0.05, ^**^ *p* <0.01, ^***^ *p* <0.001.

To evaluate the impact of Apt‐Flt3L@Lipo on the immunosuppressive microenvironment of prostate cancer (PCa), we first examined its ability to induce immunogenic cell death (ICD). Immunofluorescence staining revealed significantly elevated levels of HMGB1 and calreticulin (CRT) in tumors following Apt‐Flt3L@Lipo administration in combination with ultrasound (US), confirming robust activation of ICD (Figure 5A,B). These damage‐associated molecular patterns (DAMPs) suggest effective initiation of tumor antigen release and innate immune activation. Next, we evaluated the effect of Apt‐Flt3L@Lipo on immune cell infiltration within PCa tumors. Flow cytometry and immunofluorescence analyses showed a marked increase in DCs infiltration in tumors, with CD11c^+^ DCs rising from 5.21% to 13.4% and CD103^+^ cDC1s increasing over fourfold compared to the PBS group (Figure 5C–E). These results demonstrated that Flt3L component in Apt‐Flt3L@Lipo effectively recruited functional cDC1 to both tumor sites and tumor‐draining lymph nodes (TdLNs), thereby establishing the foundation for an in situ cDC1 vaccine. To provide spatial evidence supporting the formation of an in situ cDC1 vaccine‐like microenvironment, we performed immunofluorescence co‐localization analysis on tumor sections following systemic administration of DiR‐labeled Apt‐Flt3L@Lipo. Tumor tissues were harvested 24 h post‐injection and stained for the cDC1 marker CD103. Representative images revealed prominent spatial overlap between DiR‐labeled Apt‐Flt3L@Lipo and CD103^+^ cDC1 cells within the tumor microenvironment (Figure S8). Merged images showed clear co‐localization signals, which were further supported by line‐scan fluorescence intensity analyses demonstrating coincident peaks of CD103 and Apt‐Flt3L@Lipo signals across selected regions of interest. These results indicate that Apt‐Flt3L@Lipo preferentially accumulates in proximity to intratumoral cDC1s, providing direct spatial evidence that supports its role in establishing an in situ cDC1 vaccine‐like immune niche.

This cDC1 activation further translated into potent T cell responses. The proportion of tumor‐infiltrating CD8^+^ T cells within the CD45^+^ immune cell population increased dramatically following Apt‐Flt3L@Lipo treatment (Figure 5H; Figure S9), with significantly elevated expression of pro‐inflammatory cytokines (IFN‐γ, TNF‐α, IL‐1β, IL‐6) in the tumor microenvironment (Figure 5F). To exclude potential denominator effects caused by tumor cell loss, we further quantified the absolute number of tumor‐infiltrating immune cells. As shown in Figure 5G, Apt‐Flt3L@Lipo treatment significantly increased the absolute number of CD45^+^ immune cells per milligram of tumor tissue, indicating genuine immune cell recruitment rather than a relative proportion shift. Consistently, CD8^+^ T cells exhibited both increased absolute numbers and elevated proportions among CD45^+^ immune cells, demonstrating a true enhancement of CD8^+^ T cell infiltration. These findings collectively demonstrate that Apt‐Flt3L@Lipo successfully reprogrammed the immunosuppressive tumor microenvironment, which serves as the fundamental basis for developing an in situ cDC1 vaccination strategy.

Although Apt‐Flt3L@Lipo combined with US reduced tumor size and activated immune responses, some tumors in the Apt‐Flt3L@Lipo group continued to grow. Recent studies have reported that tumors often exploit various mechanisms to evade immune detection, one of which involves the upregulation of PD‐L1 on DCs. PD‐L1 expression on DCs can attenuate T cell activation and modulate responses to immune checkpoint blockade. In this context, we explored the PD‐L1 change on DCs and CD103^+^ cDC1 after Apt‐Flt3L@Lipo combined with US. The results indicated PD‐L1 expression on DCs, particularly CD103^+^ cDC1s, was significantly upregulated after treatment, indicating the potential for PD‐L1 blockade to restore the ability of cDC1s to activate T cells (Figure 5H; Figure S10). Furthermore, given that Apt‐Flt3L@Lipo reprogrammed the immunosuppressive microenvironment of PCa, we simultaneously observed that tumor cells in the Apt‐Flt3L@Lipo group exhibited significantly higher PD‐L1 expression compared to untreated tumors (Figure S11), while no significant difference was observed in the expression of PD‐1 on T cells in tumor and TDLN (Figure S12). These immune checkpoint molecule changes found in the post‐treatment tumor microenvironment all suggest the potential for combination with anti‐PD‐L1 therapy.

To evaluate whether Apt‐Flt3L@Lipo could enhance the antitumor immune response mediated by PD‐L1 blockade, we established a mouse primary and distant tumor model on days 0 and 5. And mice in the treatment groups received three intravenous injections of anti‐PD‐L1 antibody, as shown in Figure 5I. When combined with anti‐PD‐L1, Apt‐Flt3L@Lipo + US further inhibited the growth of both treated and distant tumors over the 30‐day observation period, whereas anti‐PD‐L1 monotherapy showed limited efficacy (Figure 5J,K). Compared to Apt‐Flt3L@Lipo + US treatment alone, the combination with anti‐PD‐L1 further improved survival (Figure 5L). Bodyweight changes across the four groups were similar during treatment (Figure S13). Moreover, immunofluorescence staining of untreated tumors demonstrated that both the Apt‐Flt3L@Lipo + US + anti‐PD‐L1 group and the Apt‐Flt3L@Lipo group activated CD8^+^ T cells in distant tumors (Figure S14). CD8 fluorescence intensity in distant tumors was 2.22 times higher in the anti‐PD‐L1‐treated group compared to the group without anti‐PD‐L1 treatment, highlighting the potential of combining Apt‐Flt3L@Lipo with immune checkpoint inhibitors for clinical applications.

Blood analysis was also carried out to examine the treatment‐associated toxicity. Typical parameters relative to the function of the liver and kidney and hematological toxicity were examined. No significant difference in the parameters of Apt‐Flt3L@Lipo group, when compared to the control group, suggesting the good tolerance of the therapy at the dose given (Figure S15).

To further substantiate the biosafety of systemic Apt‐Flt3L@Lipo administration combined with repeated ultrasound exposure, major organ histological analyses were performed. Heart, liver, spleen, lung, and kidney tissues were collected and subjected to H&E staining. No obvious tissue damage, necrosis, or inflammatory cell infiltration was observed in any examined organs compared with PBS‐treated controls (Figure S16). In parallel, TUNEL staining revealed negligible apoptotic signals in all major organs, indicating the absence of detectable treatment‐induced cell death (Figure S17). These findings are consistent with blood biochemistry and hematological analyses and collectively demonstrate that Apt‐Flt3L@Lipo combined with ultrasound exhibits favorable in vivo safety under the tested conditions.

**FIGURE 6:**
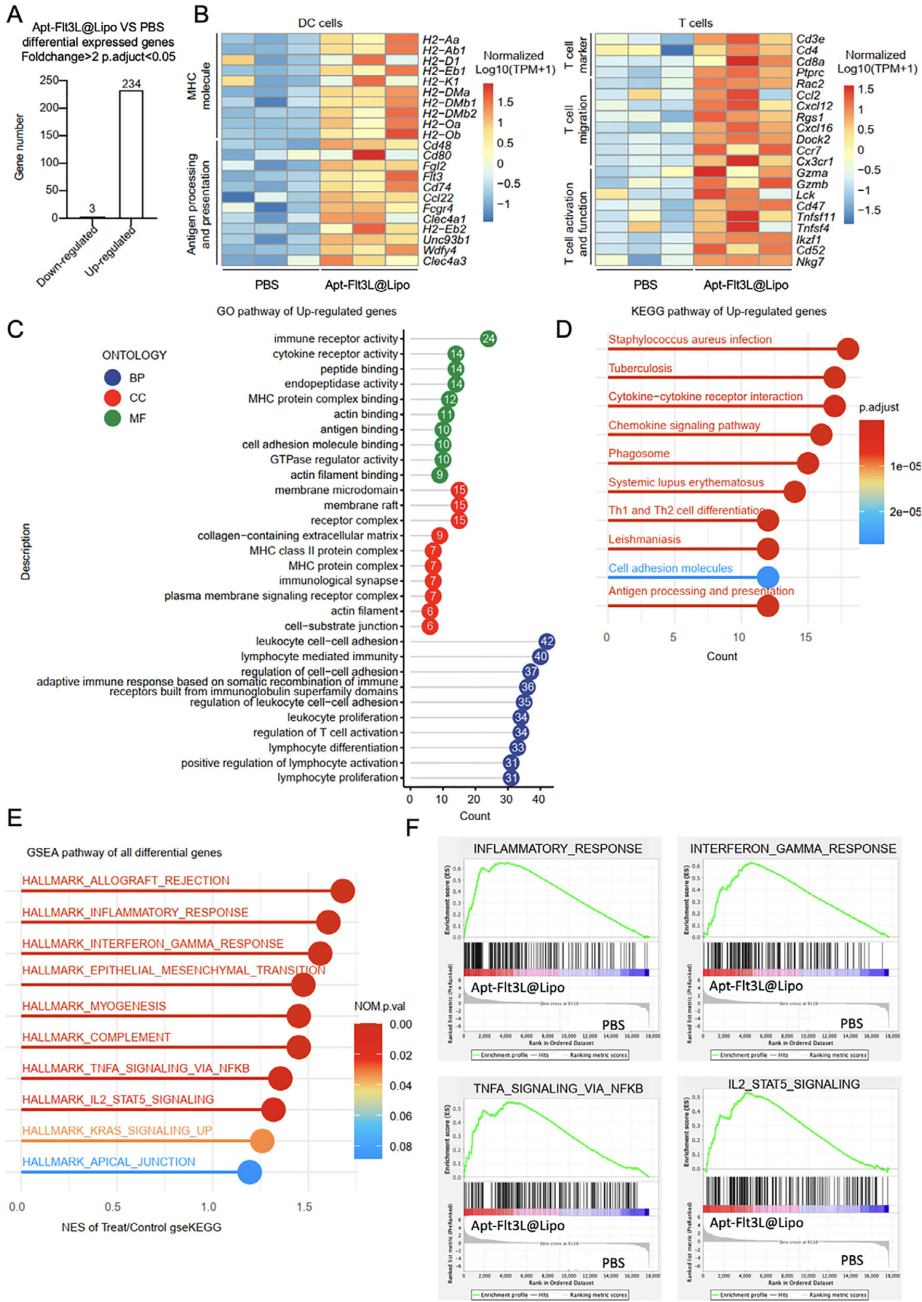
Apt‐Flt3L@Lipo induced prostate cancer‐related transcriptomic changes compared to PBS control. (A) Analysis of differentially expressed genes (DEGs) between PBS and Apt‐Flt3L@Lipo‐treated groups. The majority of DEGs were upregulated, indicating significant transcriptional changes induced by the Apt‐Flt3L@Lipo treatment. (B) Heatmap showing RNA sequencing results for DEGs associated with dendritic cells (left) and T cells (right). Genes involved in antigen presentation, MHC molecule activity, and T cell activation were significantly upregulated in the Apt‐Flt3L@Lipo group. (C) Gene ontology (GO) enrichment analysis of upregulated DEGs. Categories include biological processes (BP, blue), cellular components (CC, red), and molecular functions (MF, green), with key terms related to immune activation and antigen processing. (D) KEGG pathway enrichment analysis of upregulated genes. Top enriched pathways are shown, with dot color representing adjusted *p*‐values and dot size indicating gene count. (E) Gene set enrichment analysis (GSEA) of hallmark pathways. Upregulated pathways included inflammatory response, interferon‐gamma signaling, TNF‐alpha signaling via NF‐κB, and IL‐2/STAT5 signaling, underscoring the immunostimulatory effects of Apt‐Flt3L@Lipo. (F) Representative enrichment plots from GSEA analysis highlight robust activation of inflammatory and immune‐related pathways in the Apt‐Flt3L@Lipo group compared to PBS group.

To further elucidate and verify the underlying mechanisms of Apt‐Flt3L@Lipo treatment, we performed RNA sequencing transcriptomic analysis of post‐treatment tumors. A total of 237 differentially expressed genes (DEGs) were identified, of which 234 were significantly upregulated, 3 were downregulated (Figure 6A; Figure S18). Heatmaps of gene expression revealed significant upregulation of genes involved in antigen presentation (e.g., H2‐Ab1, Cd74), DCs function, and T cell activation (e.g., Cd8a, Gzma) (Figure 6B), further indicating the formation of in situ cDC1 vaccine in tumor. Notably, after treatment, the expression of the Flt3 gene in DCs was upregulated, demonstrating that the targeted delivery of Flt3L in this study plays a crucial role in the activation and differentiation of DCs in the tumor microenvironment. Gene Ontology (GO) (Figure 6C) and Kyoto Encyclopedia of Genes and Genomes (KEGG) (Figure 6D) functional enrichment analyses identified pathways significantly enriched among the DEGs, including antigen presentation, MHC complex assembly, immunoglobulin production, positive regulation of T cell activation, and leukocyte cell‐cell adhesion. Furthermore, GSEA demonstrated significant enrichment of pathways involved in inflammatory responses, interferon‐gamma signaling, and TNF‐alpha signaling via NF‐κB, which are critical for effective antitumor immunity (Figure 6E,F).

### 2.6 Apt‐Flt3L@Lipo Combined With SDT Enhances In Situ cDC1 Vaccine‐Mediated Anti‐Tumor Immune Responses in Patient‐Derived Ex Vivo Models

**FIGURE 7:**
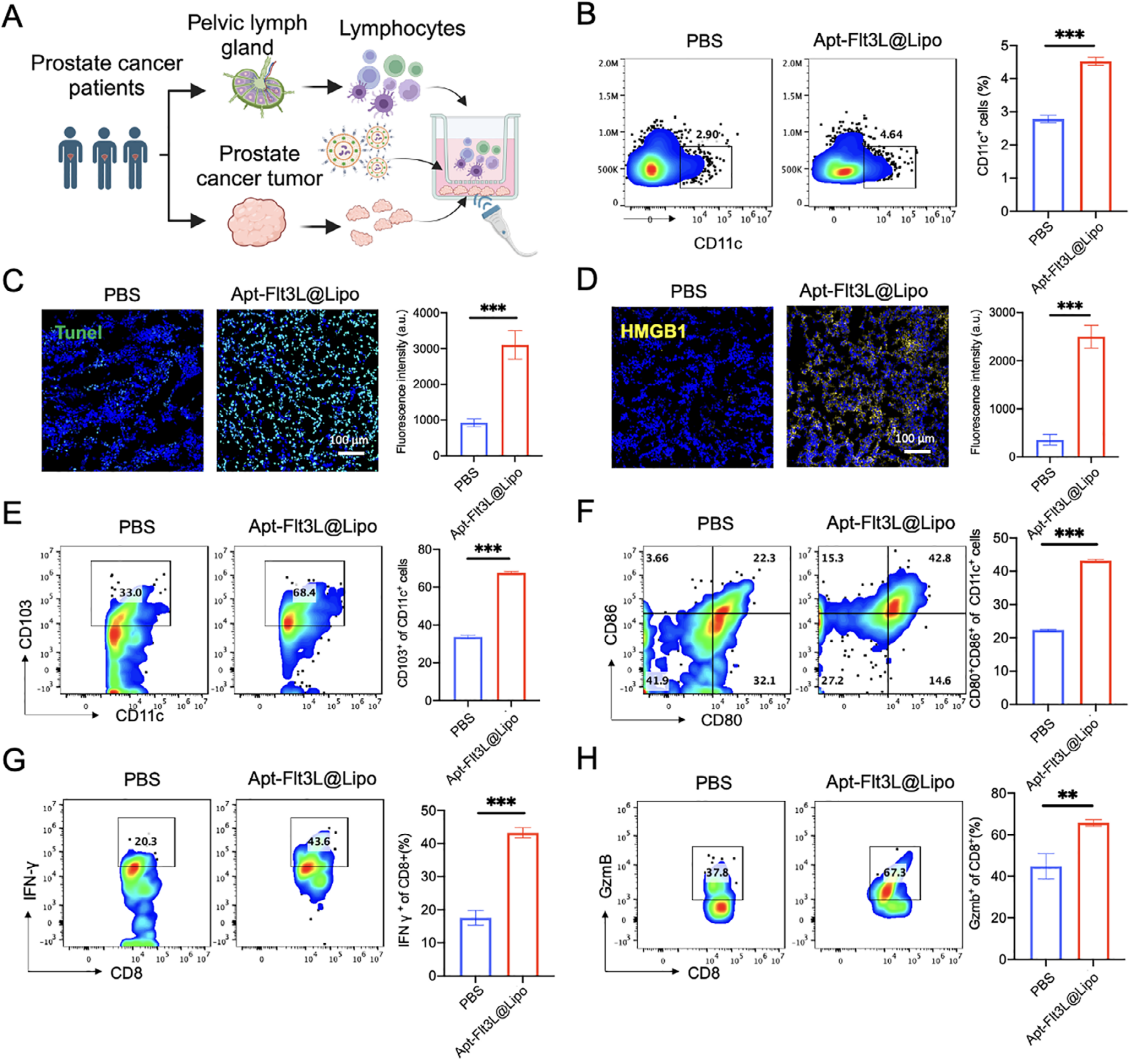
Apt‐Flt3L@Lipo enhanced responsiveness to immunotherapy in patient‐derived PCa models. (A) Schematic of a transwell experiment simulating in vivo scenario to observe DC cell migration using patient samples. (B) Flow cytometry of CD11c^+^ DC cells in the lower transwell chamber in PBS and Apt‐Flt3L@Lipo group, and their flow cytometric analysis. Immunofluorescence staining for Tunel (green) (C) and HMGB1 (yellow) (D) in tumor in PBS and Apt‐Flt3L@Lipo group and quantification analysis. Flow cytometry of cDC1 CD11c^+^CD103^+^ DC cells (E), CD80^+^CD86^+^ DC cells (F), IFN‐γ^+^CD8^+^ T cells (G), and GzmB^+^CD8^+^ T cells (H) in the lower transwell chamber in PBS and Apt‐Flt3L@Lipo group and their flow cytometric analysis. Data are presented as mean ± s.d. ^*^ *p* <0.05, ^**^ *p* <0.01, ^***^ *p* <0.001.

Next, we investigated whether the effects of Apt‐Flt3L@Lipo combined with SDT on tumors and DCs in human PCa samples were consistent with our previous findings. Matched PCa and pelvic lymph node (PLND) samples were obtained from patients (provided by Renji Hospital, Shanghai Jiao Tong University School of Medicine, China) for subsequent experimental investigations. Similar to our in vitro experiments with murine prostate cancer cells and BMDCs, we treated human prostate cancer and PLND tissues with Apt‐Flt3L@Lipo and applied US treatment. Specially, to mimic the in vivo setting, we used a transwell assay, placing tumor tissues in the lower chamber and PLND single cell suspensions in the upper chamber to observe DCs migration and activation, as shown in Figure 7A. The results demonstrated that after treatment, the proportion of CD11c^+^ DCs in the lower chamber increased from 2.78% to 4.52%, indicating enhanced DCs migration to the tumor after Apt‐Flt3L@Lipo treatment, consistent with our previous findings (Figure 7B). To further investigate the relationship between Flt3L and tumor‐infiltrating DCs, we analyzed PCa‐related data from the TCGA database. Our findings revealed that higher tumor cell proportions were associated with lower FLT3L levels, suggesting that FLT3L is predominantly derived from immune cells rather than tumor cells. Additionally, FLT3L expression showed a positive correlation with myeloid DCs infiltration, indicating that Flt3L may facilitate DCs activation and recruitment in PCa, thereby potentially contributing to anti‐tumor immunity. These results highlight the critical role of Flt3L in modulating DCs‐mediated immune responses within PCa tumor microenvironment. (Figure S19). Furthermore, H&E staining revealed that our treatment disrupted the original morphology of tumor cells and increased necrotic regions within the tumor (Figure S20). Immunofluorescence staining showed a decrease in Ki67 expression, along with an increase in TUNEL and HMGB1 levels, indicating that Apt‐Flt3L@Lipo combined with SDT inhibited tumor cell proliferation, induced cell death, and triggered ICD (Figure 7C,D; Figure S21). Similarly, we directly stimulated lymph node cell suspensions with treated tumor tissues by Apt‐Flt3L@Lipo combined with SDT. The results of flow cytometry analysis demonstrated an increase in CD103^+^ CD11c^+^ cDC1and CD80^+^ CD86^+^ DC subsets, suggesting that DCs underwent maturation and functionalization into cDC1 (Figure 7E,F). Additionally, existing T cells secreted more IFNγ and Gzmb, indicating enhanced T cell‐mediated antitumor immunity (Figure 7G,H). We concluded that the immunostimulatory activity of Apt‐Flt3L@Lipo combined with SDT is likely consistent between human and murine models. These ex vivo experiments using patients’ tumor and PLND samples demonstrated that our Apt‐Flt3L@Lipo was able to recruit and activate cDC1 for better enhancing CD8^+^ T cell cytotoxicity, showing promising potential for clinical translation.

## 3 Discussion

Prostate cancer, as a prototypical “cold tumor,” is characterized by low immunogenicity, insufficient infiltration of effector T cells, and the accumulation of immunosuppressive cells, which severely limit the efficacy of immunotherapy. In this study, bioinformatics analysis revealed that tumor‐infiltrating CD103^+^ cDC1s are significantly associated with patient prognosis (Figure 1C,D). These cells possess potent antigen processing and presentation capabilities, making them critical targets for tumor vaccine strategies. To this end, we developed a combinatorial strategy that integrates sonodynamic therapy (SDT) with the dendritic cell growth factor Flt3L to activate an “in situ cDC1 tumor vaccine” in vivo. Compared with traditional ex vivo‐prepared DC vaccines (such as the FDA‐approved Sipuleucel‐T), this in situ activation approach offered several distinct advantages. On the one hand, SDT induced by ultrasound effectively triggered ICD in tumor cells, leading to the robust release of patient‐specific tumor antigens and damage‐associated molecular patterns (DAMPs), thereby enhancing the diversity and immunogenicity of the antigen repertoire. On the other hand, the spatiotemporally controlled release of Flt3L selectively expanded and activated the cDC1 subset, boosting its capacity for antigen cross‐presentation and chemotactic recruitment, ultimately eliciting a potent CD8^+^ T cell‐mediated immune response. This “in situ induction plus endogenous activation” model overcame the major limitations of traditional DCs vaccines, including limited antigen spectrum, complex manufacturing processes, functional deterioration during in vitro manipulation, and poor viability after reinfusion. As a result, this strategy significantly improved the clinical translational potential of DCs‐based immunotherapies.

From a formulation perspective, this study employed a uniquely designed amphiphilic delivery system capable of efficiently co‐delivering hydrophilic Flt3L and hydrophobic chlorin e6 (Ce6). The system was further functionalized with an EpCAM‐targeting aptamer to enable precise delivery to prostate cancer tissue. This platform not only enhanced the biodistribution and tumor accumulation of therapeutic agents but also achieved synchronized, ultrasound‐triggered, spatiotemporal release of both Ce6 and Flt3L. In addition, both the liposomal formulation and nucleic acid aptamer exhibit strong translational advantages, including ease of manufacturing, excellent batch‐to‐batch consistency, and long‐term storage stability (Figure 1D). These features markedly improved the formulation's potential for clinical chemistry, manufacturing, and control (CMC) translation and lay the foundation for further clinical development. This study presents a novel strategy for immunotherapy in PCa. Although its efficacy has been demonstrated in patient‐derived ex vivo explant and transwell validation models, further validation using clinical samples and large animal models is still required to comprehensively evaluate its therapeutic effectiveness and safety in clinical applications. In terms of targeting efficacy, multi‐target strategies (EpCAM combined with prostate membrane‐specific antigen) or alternative targeting methods that do not rely on EpCAM can be explored for PCa.

In summary, this study employed sonodynamic therapy to induce an in situ cDC1 vaccine, thereby reprogramming the antitumor immune landscape within the prostate tumor microenvironment. This strategy offered a promising new avenue for precision immunotherapy in prostate cancer and provided a theoretical foundation for extending immunotherapeutic applications to other low‐immunogenic solid tumors.

## Author Contributions

X.L., W.X., J.P., and Y.Y. contributed to the conceptualization of the study. The methodology was developed by X.L. and J.W., while validation was carried out by X.L. and Yu Yang. Formal analysis was performed by J.W., X.L., W.X., J.P., and Y.Y. Investigation was conducted by X.D., L.T., W.S., X.L., and J.W. Data curation was handled by X.D., L.T., X.L., and J.W. The original draft was written by X.L. and Jiayi Wang, and the review and editing were completed by X.L., W.X., J.P., and Y.Y. Visualization was carried out by X.L. and J.W. Supervision was provided by X.L., W.X., J.P., and Y.Y. Project administration was managed by X.L. and Y.Y., and funding acquisition was secured by X.L., W.X., J.P., and Y.Y.

## Funding

This work was supported by the National Key Research and Development Program of China (No. 2022YFA1206500), the Fundamental Research Funds for the Central Universities (No. 2020JCPT02), the National Natural Science Foundation of China (No. 22277072, 32401161), the Shuguang Program of Shanghai Education Development Foundation and Shanghai Municipal Education Commission (No. 24SG20), Shanghai Oriental Talents (No. QNWS2024055), the Natural Science foundation of Shanghai, China (No. 24ZR1462700, 25ZR1402322), the Science and Technology Development Fund of Pudong Health Bureau of Shanghai (No. PKJ2024‐Y40).

## Conflicts of Interest

The authors declare no conflicts of interest.

## Supporting information




**Supporting File**: advs74518‐sup‐0001‐SuppMat.docx.

## Data Availability

The data that support the findings of this study are available from the corresponding author upon reasonable request.
