# Supplementary material for: Aptamer‐Engineered Liposomal Platform Enables in Situ cDC1 Vaccination to Potentiate Immunotherapy in Prostate Cancer
Source: Adv Sci (Weinh). 2026 Feb 24:e22169. Online ahead of print. doi: 10.1002/advs.202522169 (PMC13326002; doi:10.1002/advs.202522169)
Supplement: Supplementary file 1 — Supporting File: advs74518‐sup‐0001‐SuppMat.docx. [file ADVS-9999-e22169-s001.docx]

**Supporting information**

**Aptamer-engineered liposomal platform enables in situ cDC1 vaccination to potentiate immunotherapy in prostate cancer**

Jiayi Wang^1,2^, Xuan Wang^1^, Xinfeng Dai^1,3^, Linxin Tian^1^, Wencheng Shen^1,4^, Xueliang Liu^1,2^*, Wei Xue^1^*, Jiahua Pan^1^*, Yu Yang^1,2^*

1. Institute of Molecular Medicine (IMM) and Department of Urology, Renji Hospital, School of Medicine, Shanghai Jiao Tong University, Shanghai, 200127, China;

2. Punan Branch of Renji Hospital, Shanghai jiaotong University School of Medicine, Shanghai 200125, China.

3. School of Life Science, Shanghai University, Shanghai, 200444, China;

4. College of Chemistry and Materials Science, Shanghai Normal University, Shanghai 200234, China;

Corresponding authors: yuyang@shsmu.edu.cn (Yu Yang); panjiahua@renji.com (Jiahua Pan); uroxuewei@163.com (Wei Xue); xuelianghc@163.com (Xueliang Liu)

**Methods**

**Materials, cell, and animal**

DPPC, DOTAP and cholesterol were purchased from AVT Pharmaceutical Tech Co., Ltd; CCK-8, Hoechst 33342, Dulbecco’s modified Eagle’s medium (DMEM), fetal bovine serum (FBS), 1640 medium, penicillin, streptomycin and trypsin were obtained from Sigma-Aldrich (St. Louis, USA). Ce6 was purchased from Absin Bioscience Inc. Recombinant mouse and human Flt3L were purchased from R&D Systems (Minneapolis, MN, USA). EpCAM aptamer were synthesized and purified by Huzhou Hippo Biotechnology Co. Ltd. (Huzhou, China). Calcein AM, Annexin/PI kit and DCFH-DA probe were purchased from Beyotime Biotechnology Co., Ltd (Shanghai, China). The αPD-L1 antibody was purchased from Bioxcell, Inc.; Antibodies against CD11c, CD103, CD80, CD86, CD3, CD4, CD8, CD69, CD44, CD62L, TNF-a IFN-γ, Ki-67, Gzmb and TNF-α were obtained from BD Biosciences.

RM1 cells were obtained from the American Technical Center for Biological Reference Collection (ATCC) and cultured in DMEM medium containing 10% FBS and 1% penicillin-streptomycin (double antibody) (37 ℃, 5% CO2), respectively. Bone marrow-derived dendritic cells (BMDCs) were obtained from the tibiae and femurs of male C57BL/6 mice. The BMDCs were cultured in RPMI 1640 medium containing 10% FBS, 1% penicillin-streptomycin, 1% β-mercaptoethanol, 20 ng/mL GM-CSF and 10 ng/mL IL-4.

C57BL/6J mice (male, age 6 to 7 weeks) were obtained from Shanghai Laboratory Animal Center (China). All animal experiments were performed under the guidelines evaluated and approved by the ethics committee of Institutional Animal Care and Use Committee of Ren Ji hospital, Shanghai Jiao Tong University.

**Human subject**

These studies were approved by the Ethics Committees of Renji Hospital, Shanghai Jiaotong University School of Medicine, and the ethics number is KY2023-112-C. All patients were given informed consent for sample collection.

**Preparation and characterization of Apt-Flt3L@Lipo**

Firstly, DPPC (25 mg), DOTAP (25 mg), cholesterol (25 mg), and ce6 (5 mg) were mixed in 1 mL chloroform. Subsequently, DPPC, DOTAP, cholesterol, and ce6 were added to a chloroform-methanol (2.5mL+1.5mL) mixture in a 5:1:1:1 ratio. The solvent was removed by rotary evaporator (65 rpm, 30 min), and the resulting film was hydrated with 4 mL of aqueous or PBS solution of Flt3L (150 rpm, 25°C, 2 h). The suspension was then filtered through 400 nm, 200 nm, and 100 nm membranes. Next, 400 µL of 10 µM cholesterol-modified EpCAM aptamer was added (25°C, 2 h), allowing the aptamer to conjunct to the surface of the liposomes via the hydrophobic interaction of cholesterol. The Apt-Flt3L@Lipo particles were then collected and stored at 4°C for future use.

Confirmation of component Apt-Flt3L@Lipo: Liposomes were prepared using DiR-labeled lipids, FAM-labeled EpCAM aptamer and ce6 according to the aforementioned procedure. The UV–vis–NIR absorbance spectrum was measured using a microplate reader, and *in vitro* imaging of the liposomes was performed (Ce6: Ex/Em = 400/650 nm; DiR: Ex/Em = 633/760 nm; FAM: Ex/Em = 490/520 nm) using the IVIS system.

The hydrodynamic diameters, zeta potential and PDI of Apt-Flt3L@Lipo were examined by DLS using the ZetaSizer NanoZS instrument (Malvern, Worcestershire, UK). The shape and structure of Apt-Flt3L@Lipo were characterized using TEM (je -2100, Tokyo, Japan) and SEM (TM-1000, Hitachi, Tokyo, Japan).

**Singlet oxygen release of Apt-Flt3L@Lipo**

100 μL Apt-Flt3L@Lipo was added to aqueous solution of singlet oxygen sensor green reagent (SOSG) (50 μL, 20 μM), respectively, and then the mixture was treated with or without US (10W, 50%) for 1, 2, 4 min, followed by measuring the 'O2 by a microplate reader. Green fluorescence of SOSG (Ex/Em = 504/525 nm) was detected. 'O2 generation of US-triggered liposome in cells was measured by 2ʹ,7ʹ- dichlorodihydrofluorescein diacetate (DCFH-DA). RM1 cells were seeded in 12-well plates and cultured for 12 h, followed by treatment with Lipo/Apt@Lipo/Apt@Lipo_ce6_ for 4 h, then treated with ultrasound (10W, 50%), observed under confocal laser scanning microscope (LCM).

***In vitro* evaluation of tumor apoptosis**

RM1 cells were seeded into 96-well plates (1 × 10^4^ cells/well) and incubated for 24 hours. The cells were then treated with PBS, Lipo, Lipo_ce6_, and Apt@Lipo, with or without US (10W, 50%) for 2 minutes. Cell viability was subsequently measured using the standard CCK-8 assay. Under the same treatment groups and conditions, apoptosis following Apt@Lipo + US was evaluated using Annexin-PI and Calcein-AM kits, then analyzed by flow cytometry and LCM.

**Tumor targeting of Apt@Lipo**

In the *in vitro* experiments, RM1 cells (1 × 10^4^ cells per well) were seeded onto coverslips in a 24-well plate. After 24 hours of incubation, the cells were treated with Lipo, DNA-Flt3L@Lipo, or Apt-Flt3L@Lipo for 0.5, 1, and 2 hours, respectively. The cells were then washed twice with PBS, and the fluorescence levels of ce6 within the cells were observed using LCM.

In the *in vivo* experiments, DNA-Flt3L@Lipo and Apt-Flt3L@Lipo were prepared by adding DiR into the liposomal formulations during rotary evaporation. RM1 tumor-bearing mice were established by subcutaneous injection of RM1 cells (1 × 10^6^ cells/mouse) into male C57BL/6J mice. When the tumors reached approximately 50 mm³, the mice were randomly assigned to two treatment group. Once the tumor volumes reached 200 mm³, different drugs were administered via tail vein injection, and the fluorescence intensity of DiR was monitored at 1, 2, 4, 8, 12, and 24 hours using an IVIS system and gel documentation system. After the final time point, the mice were sacrificed, and *ex vivo* imaging of the tumors was performed using IVIS. The fluorescence intensity of DiR within the tumor tissue was further analyzed by LCM.

**Apt-Flt3L@Lipo stimulates maturation and migration of DCs *in vitro***

To analyze the maturation of DCs *in vitro*, immature bone marrow-derived dendritic cells (BMDCs) from male C57BL/6 mice were seeded into 24-well plates at a density of 5 × 10^5^ cells per well. The cells were then treated with PBS, free Flt3L, or Flt3L@Lipo for 24 hours. Following treatment, the BMDCs were stained with antibodies against CD11c, CD40, CD80, CD86, MHCII, and CD103, and analyzed by flow cytometry to assess maturation and differentiation.

Next, RM1 cells were subjected to US (10W, 50%) for 2 minutes, followed by centrifugation at 2000 rpm for 10 minutes. The supernatant was collected and added to BMDCs, which were incubated with PBS, Apt-Flt3L@Lipo (no ce6), Apt@Lipo, Flt3L@Lipo, and Apt-Flt3L@Lipo for 24 hours. Flow cytometry was performed on the BMDCs using the same panel of markers to assess maturation and differentiation.

For the simulation of *in vivo* conditions, we used approximate 200 mm³ tumor-bearing mice, which were sacrificed to collect tumors and ipsilateral inguinal lymph nodes. Tumors were cut into small pieces and placed in the lower chamber of an 8μm transwell system, while the lymph nodes were processed into single-cell suspensions and placed in the upper chamber， incubated in DMEM medium for 24 hours. After incubation, the cells were stained with antibodies for CD11c, CD45, CD103, CD8, IFN-γ, TNF-α, and Granzyme B. The cells were washed twice with DPBS and analyzed by flow cytometry to evaluate immune cell migration and activation.

***In vivo* anti-tumor effects**

RM1 tumor-bearing mice were established by subcutaneously injecting RM1 cells (1×10^6^ cells/each) into male C57BL/6J mice. When the tumors reached approximately 150 mm³ after 8 days, the mice were randomly assigned to five treatment groups, with at least nine mice per group. Mice in each group received 100 µL of the respective liposomal formulation (4 μg Flt3L per mouse) via intravenous injection on days 8, 10, and 12. Four hours after each injection, the mice underwent US (10W, 50%, 2 min). Tumor size (calculated as V = length × width^2^ / 2) and body weight were monitored every other day for four consecutive weeks, with survival tracked for up to 60 days. On day 20, three mice from each group were randomly selected for sacrifice. Tumors, tumor-draining lymph nodes, and blood samples collected via orbital extraction were harvested for further analysis.

**Flow cytometry analysis of *in vivo* immune response.**

At day 20, tumors and lymph nodes were excised from mice and prepared into single cell suspensions. Cells from lymph nodes were firstly blocked by 0.5% BSA (in DPBS), then stained with CD11c, CD80 and CD86 at 4°C for 30 min to determine immune response by flow cytometry assay.

To investigate the tumor-infiltrating DCs, tumors were cut into small pieces then incubated with digestion solution (DMEM medium containing 1 mg/mL collagenase IV, 1 mg/mL collagenase I, 1 mg/mL hyaluronidase and 0.2 mg/mL DNase I). Then, single cell suspension was prepared and centrifuged, followed by resuspending the cell pellet in 50% Percoll. This cell suspension was overlaid onto 70% Percoll (in DPBS) and centrifuged for 30 min. Lymphocytes were collected at the interface between the discontinuous Percoll gradient. Then, cells were blocked by 0.5% BSA (in DPBS) and stained with CD4, CD8, CD11c, CD80, CD86, CD103, PD-1 and PD-L1. Besides, immunofluorescence staining of CD8 and CD103, the activation of cytotoxic T lymphocytes (CTLs) and cCD1 in tumor were also analyzed.

Meanwhile, to analyze the population of IFN-γ, Granzyme B and TNF-a producing CD8^+^ T cells, cells were also stained by CD3, CD8, IFN-γ, TNF-a and Granzyme B. After that, cells were washed with DPBS twice and analyzed by flow cytometry.

**Tumor RNA sequencing and gene expression analysis**

At day 20 in the treatment period, we quickly excised tumors and frozed them with liquid nitrogen (n=3). The mRNA samples of the PBS group and Apt-Flt3L@Lipo group were used for RNA-seq (BGI, Shenzhen, China). We conduct differential expression analysis using the DESeq2 package and proceed with Gene Ontology (GO) and Kyoto Encyclopedia of Genes and Genomes (KEGG) enrichment analyses for the significantly differentially expressed genes. Subsequently, we employ the ggplot2 and pheatmap packages for visualization purposes.

***In vivo* evaluation of ICD**

At day 20 in the treatment period, tumor tissues were collected from the sacrificed mice fixed with 4% paraformaldehyde (n=3). Then the tumor tissues were embedded in OCT cryogel for cryosectioning. The specific steps were as follows: (1) Tumor tissue sections were placed on slides and washed three times with PBS to 10 remove excess OCT; (2) Tumor sections were closed with 5% BSA-PBS for 1 h; (3) Each slice was incubated with 100 µL of CRT and primary antibody at 4 ℃ overnight and then washed three times repeatedly with PBS for 5 min each time; (4) Each slice was incubated with 100 µL of secondary antibody for 2 h at room temperature and then washed three times repeatedly with PBS for 5 min each time; (5) Each sample was incubated with 200 µL of hoechest at room temperature for 15 min and then washed once with PBS for 5 min; (6) Each slice was sealed with 20 microliters of sealer and analyzed by confocal microscope imaging. For evaluation of HMGB1 in mice tumor and patient PCa samples, tumor sections were treated with an HMGB1 antibody. Next, *in vivo* assessment of HMGB1 release was performed using LCM.

**Analyze of cytokine in the tumor**

For male C57BL/6 mice after treatment at day 20, their tumor tissue of the PBS group and Apt-Flt3L@Lipo group were collected. The tumor tissue was homogenized on ice and centrifuged at 12000 rpm for 25 min at 4 oC, to remove any insoluble material. The levels of IFN-γ, IL-1β, IL-6 and TNF-ɑ in tumor homogenate supernatants were measured by ELISA kits according to the manufacturer’s instructions.

**Blood tests of treated mice**

At day 20 in the treatment period, we performed orbital venous plexus blood collection in mice from the PBS and Apt-Flt3L@Lipo groups. Whole blood samples were stored at room temperature for 2 hours, followed by centrifugation at 3,000 rpm for 15 minutes at 2-8°C to obtain the supernatant. Whole blood was used for routine hematological analysis, while serum was used to assess liver and kidney function.

**Apt-Flt3L@Lipo combined with aPD-L1 inhibits distant tumor**

For the initial tumor inoculation, 1×10⁶ RM1 cells were subcutaneously injected into the right flank of male C57BL/6J mice. Five days later, an additional 1×10⁶ RM1 cells were subcutaneously injected into the left flank to establish a distant tumor model. Three days after the second inoculation, the tumor-bearing mice were randomly assigned to four groups (n = 6): PBS, αPD-L1, Apt-Flt3L@Lipo, and Apt-Flt3L@Lipo + αPD-L1. Apt-Flt3L@Lipo and αPD-L1 were administered via tail vein injection in the respective groups on days 8, 10, and 12. Four hours after drug administration, the primary tumor on the right flank was subjected to US (10W, 50%, 2 min). Tumor size and body weight were monitored for up to 4 weeks, with survival observed for up to 60 days.

**Patient sample used for DCs maturation and migration validation**

PCa tissue samples were collected from patients undergoing radical prostatectomy at Renji Hospital, Shanghai Jiao Tong University School of Medicine. Intraoperative frozen section and HE staining confirmed malignancy in the prostate tumor tissue. Simultaneously, PDLN biopsies were collected, and intraoperative frozen section and HE staining verified the absence of tumor metastasis. The tumor and lymph node tissues were processed to analyze the changes in DCs subsets and assess their migration capacity towards the transwell lower chamber post-treatment.

**Statistical analysis**

Statistical analysis was performed using GraphPad Prism 9.0 software (La Jolla CA). All data were expressed as mean ± standard deviation and statistically analyzed using Student t-test. Differences between the two groups were determined to be significant at *P < 0.05 and highly significant at **P < 0.01 and ***P < 0.001.

**
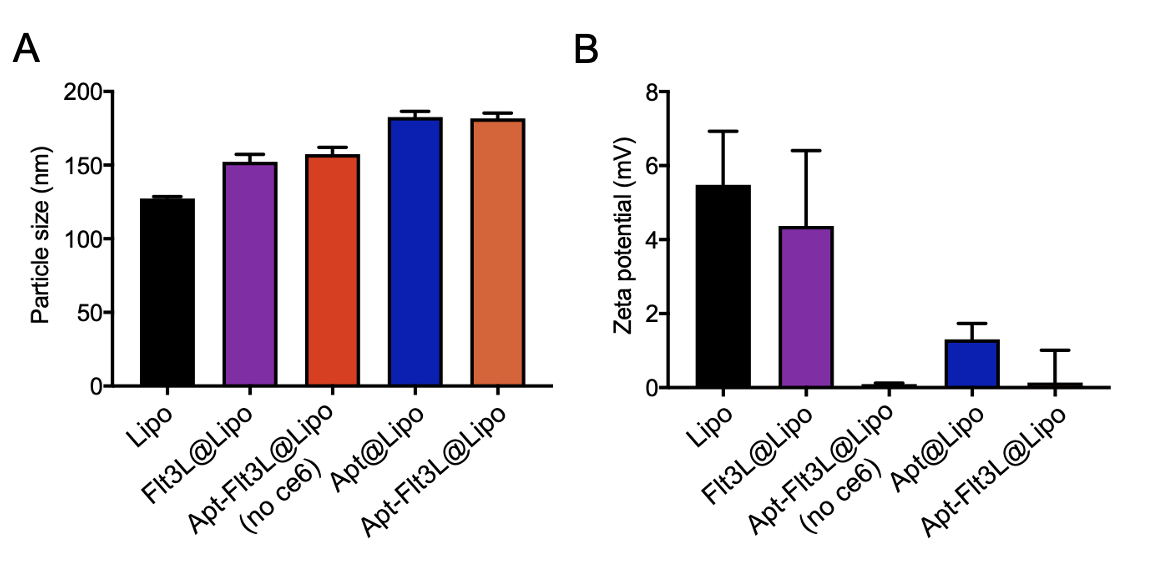
**

**Figure S1.** (A) Comparison of particle size across different formulations, including Lipo, Flt3L@Lipo, Apt-Flt3L@Lipo (without Ce6), Apt@Lipo, and Apt-Flt3L@Lipo. (B) Zeta potential measurements of Lipo, Flt3L@Lipo, Apt-Flt3L@Lipo (without Ce6), Apt@Lipo, and Apt-Flt3L@Lipo, demonstrating surface charge variations indicative of successful aptamer and Flt3L incorporation.

**
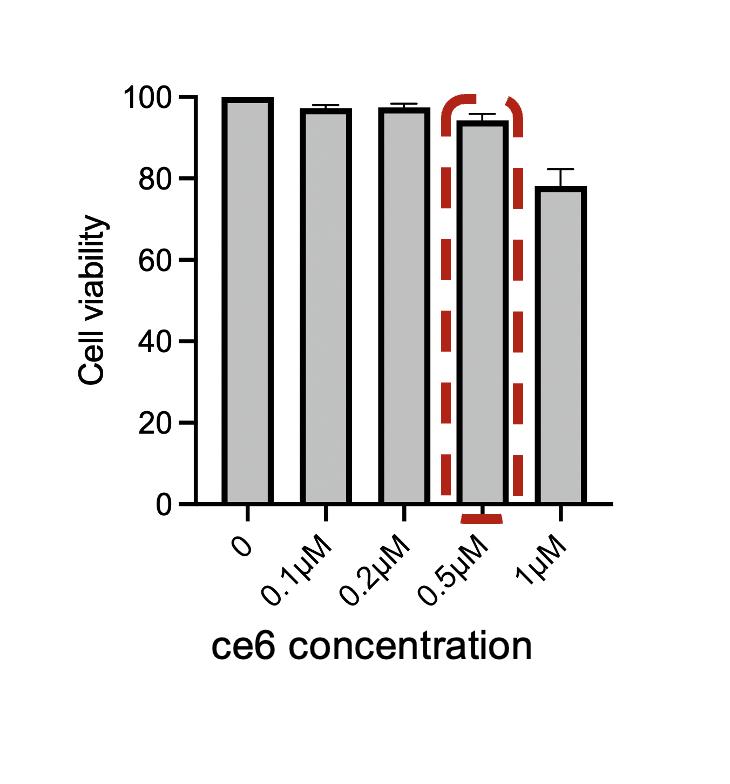
**

**Figure S2.** The effect of liposomes loaded with different concentrations of Ce6 on cell viability.


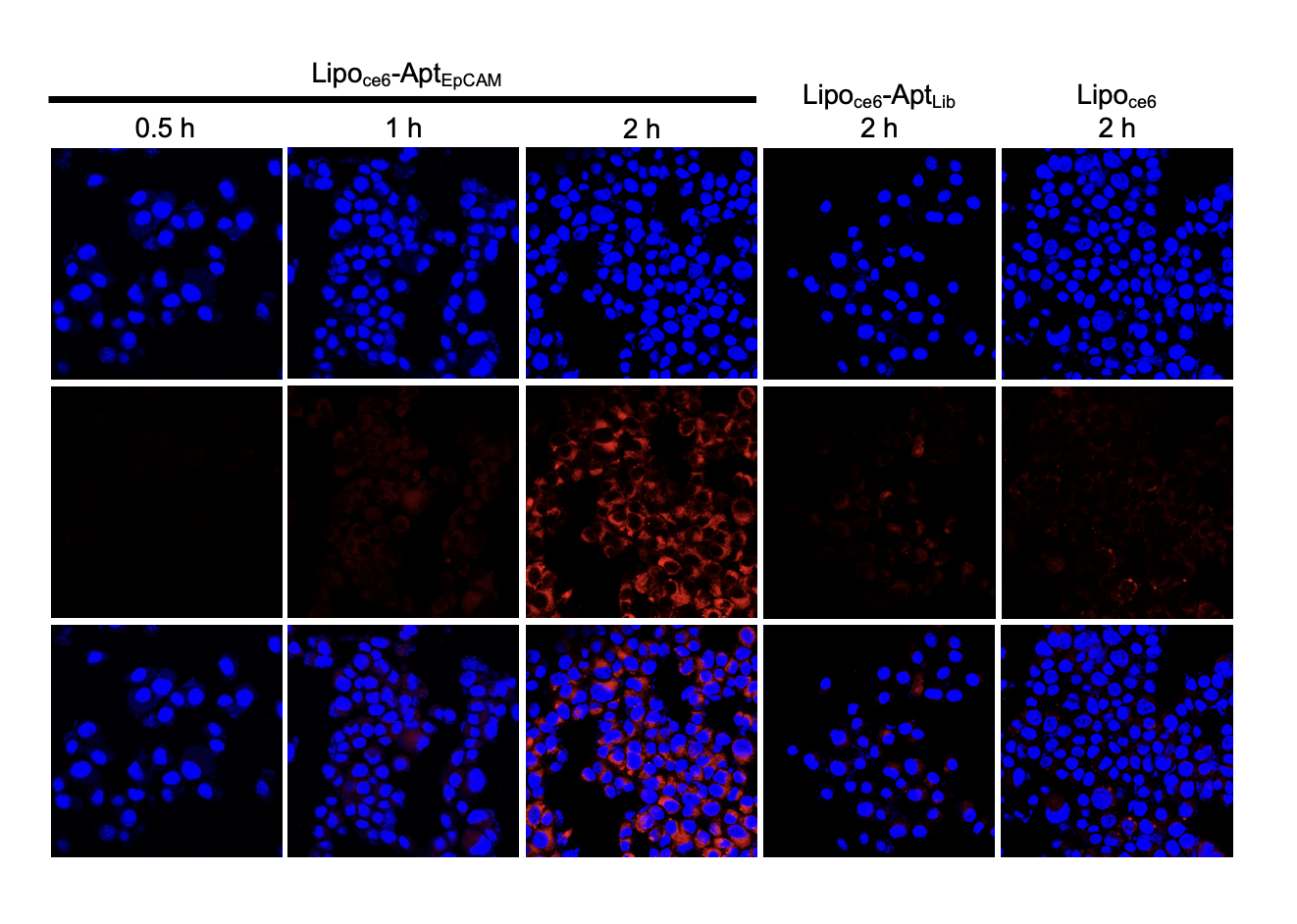


**Figure S3.** Representative confocal fluorescence images of RM1 cells treated with Lipo_ce6_, Lipo_ce6_-Apt_Lib_ (Random sequence of the DNA), Lipoce6-Apt_EpCAM_ for different times.

**
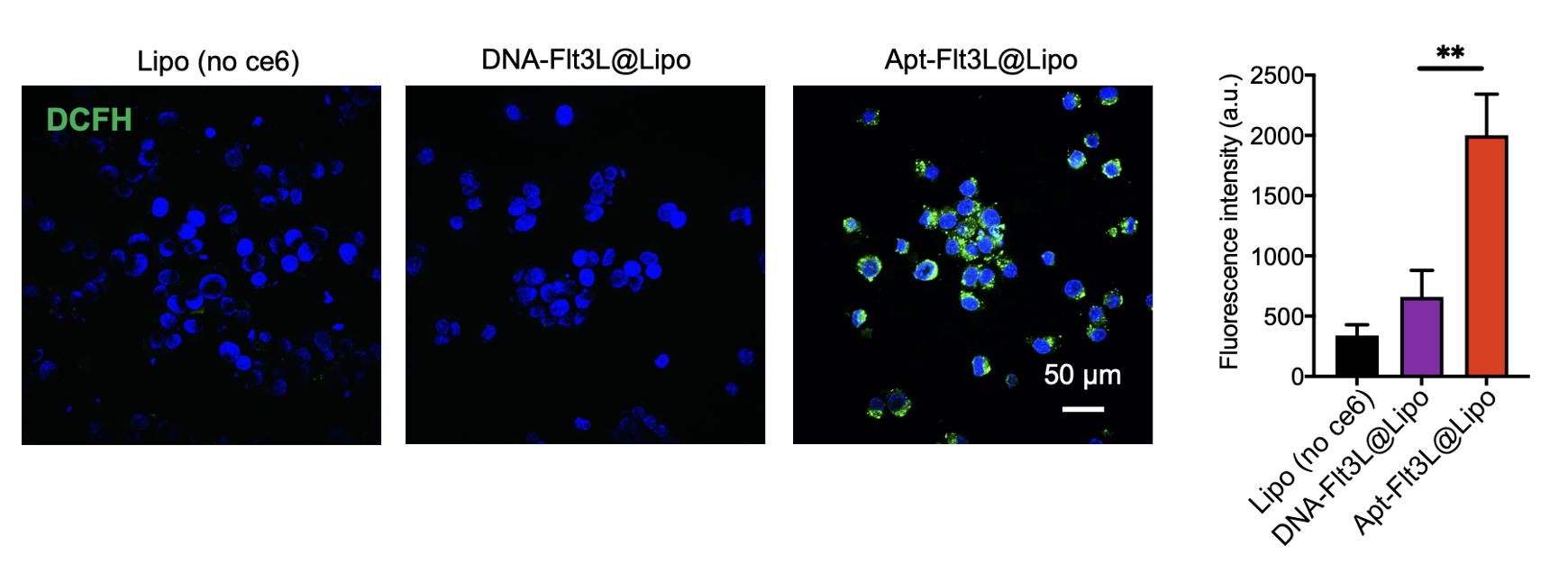
**

**Figure S4.** Immunofluorescence staining and quantification analysis showing ROS generation evaluation using DCFH probe imaging in RM1 prostate cancer cells treated with Apt-Flt3L@Lipo combined with US

**
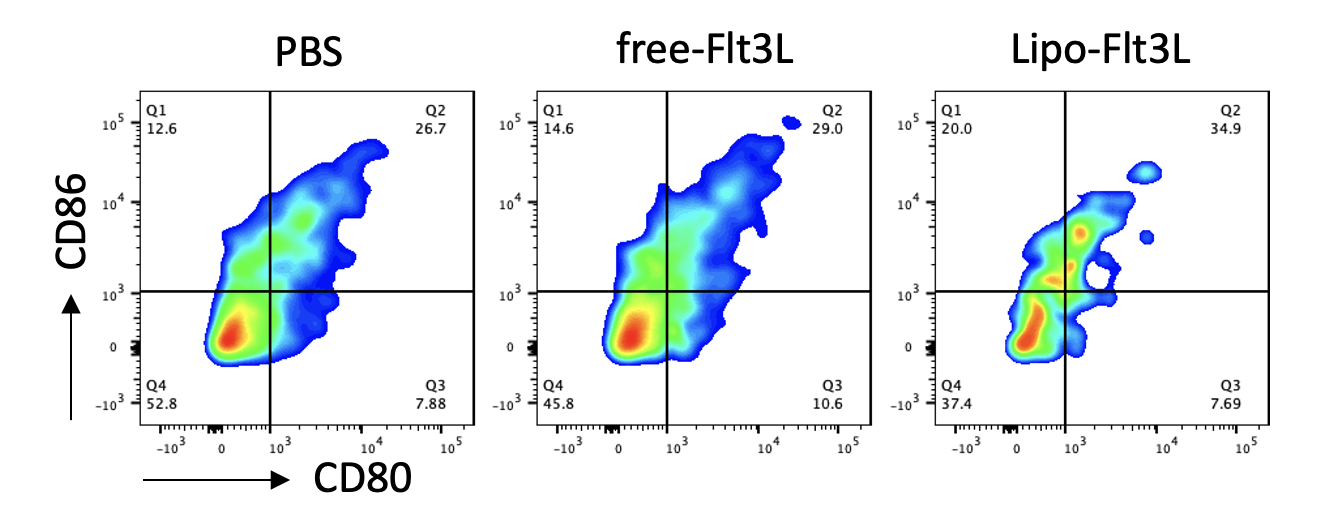
**

**Figure S5.** Flow cytometry analysis of CD80^+^CD86^+^ cells in CD11c^+^ BMDCs co-incubated with PBS, free-Flt3L and Lipo-Flt3L.

**
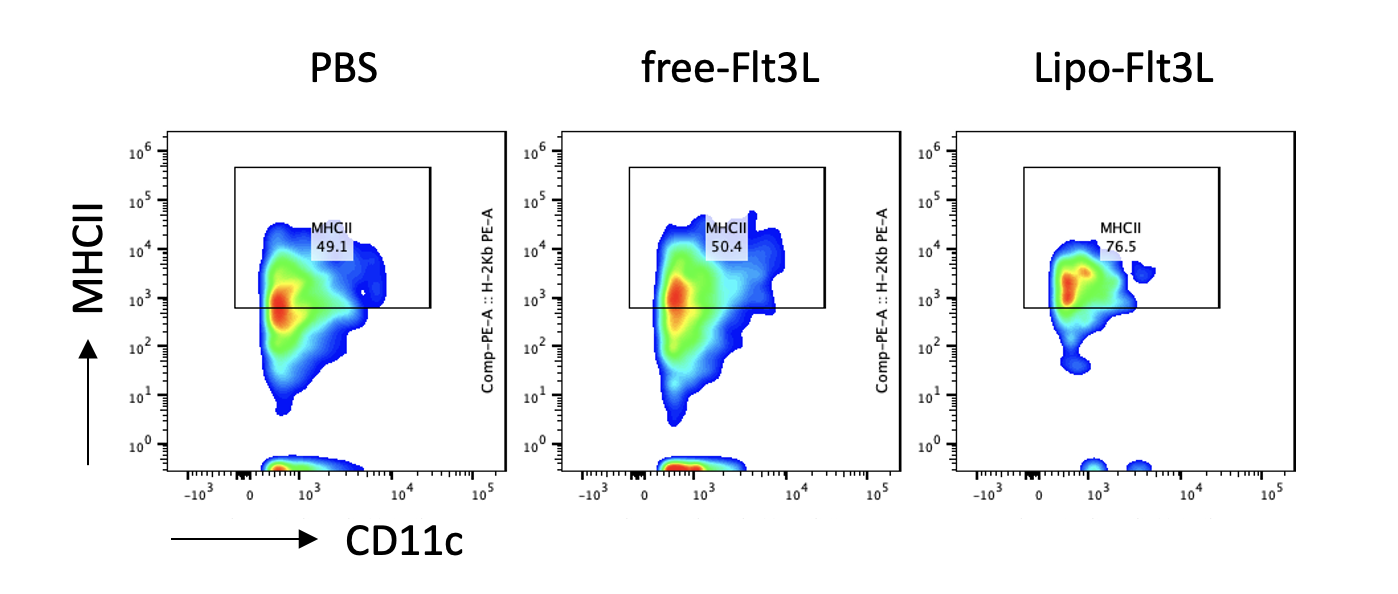
**

**Figure S6.** Flow cytometry analysis of MHCII^+^CD11c^+^ cells in BMDCs after incubation with PBS, free-Flt3L and Lipo-Flt3L.

**
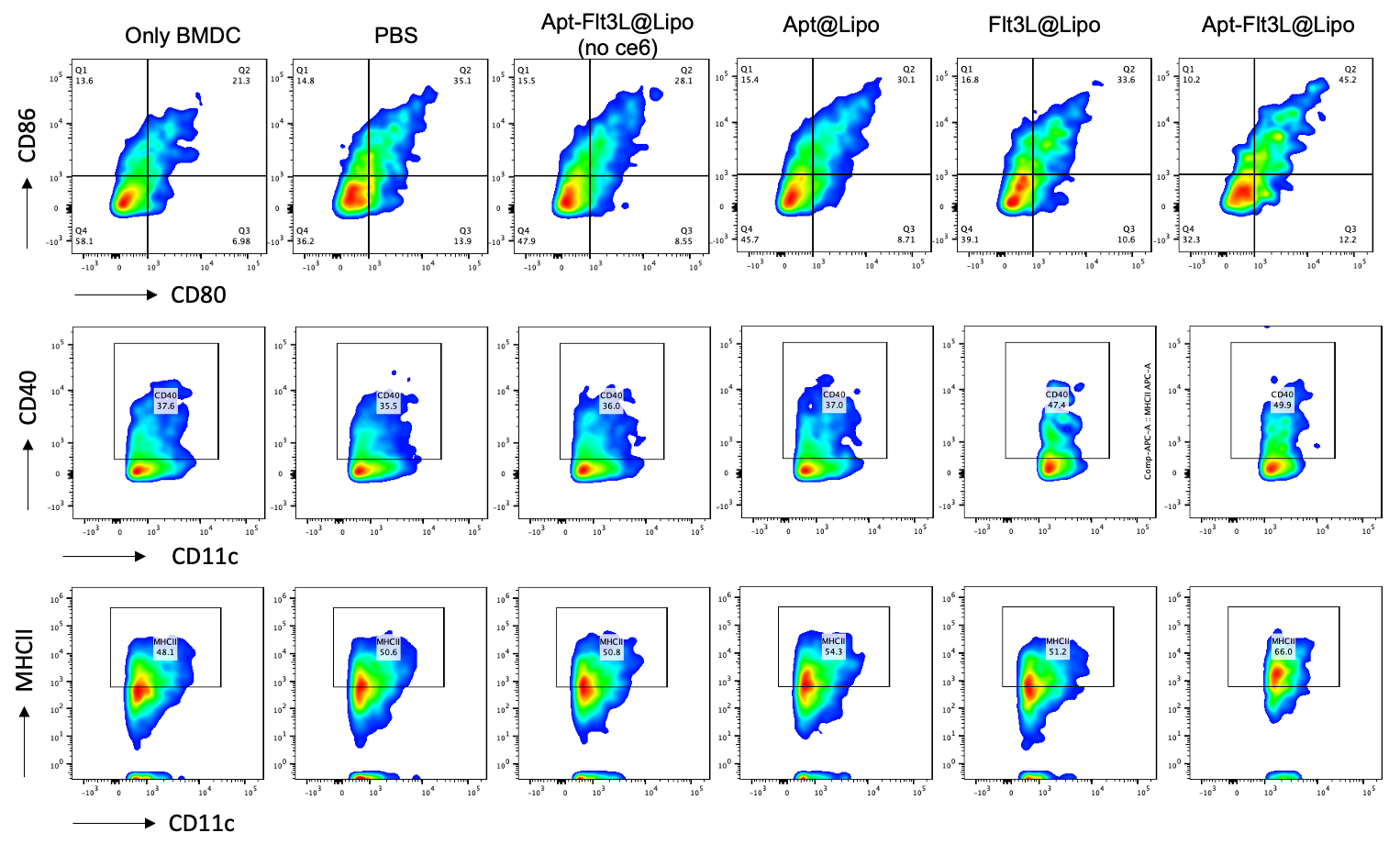
**

**Figure S7.** Flow cytometry analysis of CD80^+^CD86^+^ cells, CD40^+^CD11c^+^ cells and MHCII^+^CD11c^+^ cells in RM1-OVA tumor treated with Apt-Flt3L@Lipo and US.


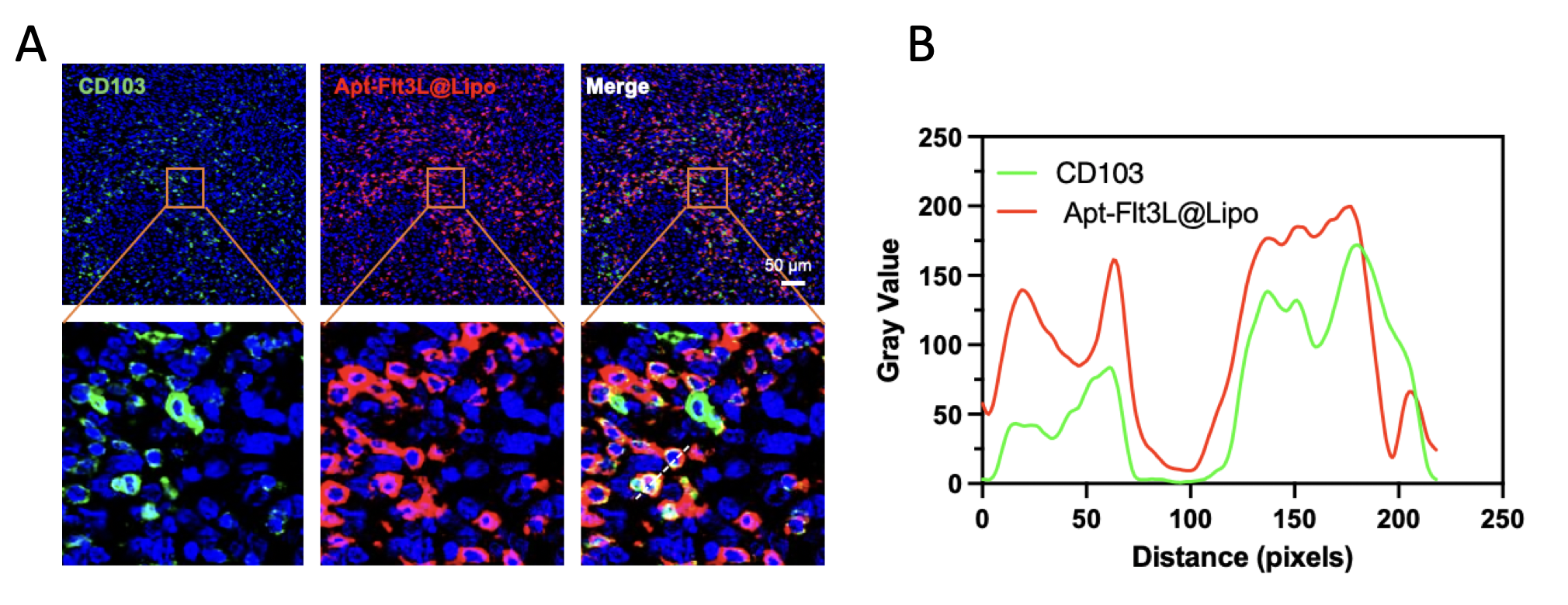


**Figure S8.** (A) Representative immunofluorescence images of tumor sections collected from tumor-bearing mice 24 h after intravenous administration of DiR-labeled Apt-Flt3L@Lipo. Tumor sections were stained for the cDC1 marker CD103 (green), while Apt-Flt3L@Lipo was visualized by DiR fluorescence (red). Merged images reveal prominent spatial overlap between Apt-Flt3L@Lipo and CD103⁺ cDC1 cells within the tumor microenvironment, as highlighted in the magnified regions. Scale bar, 50 μm. (B) Line-scan fluorescence intensity profiles across selected regions of interest further demonstrate coincident signal peaks of CD103 and Apt-Flt3L@Lipo, indicating non-random spatial association.


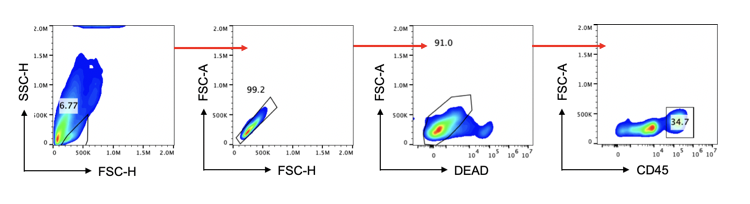


**Figure S9.** Flow cytometry gating strategy included sequential selection of cells based on FSC/SSC, singlet discrimination, live cell gating, and CD45⁺ immune cell identification.

**
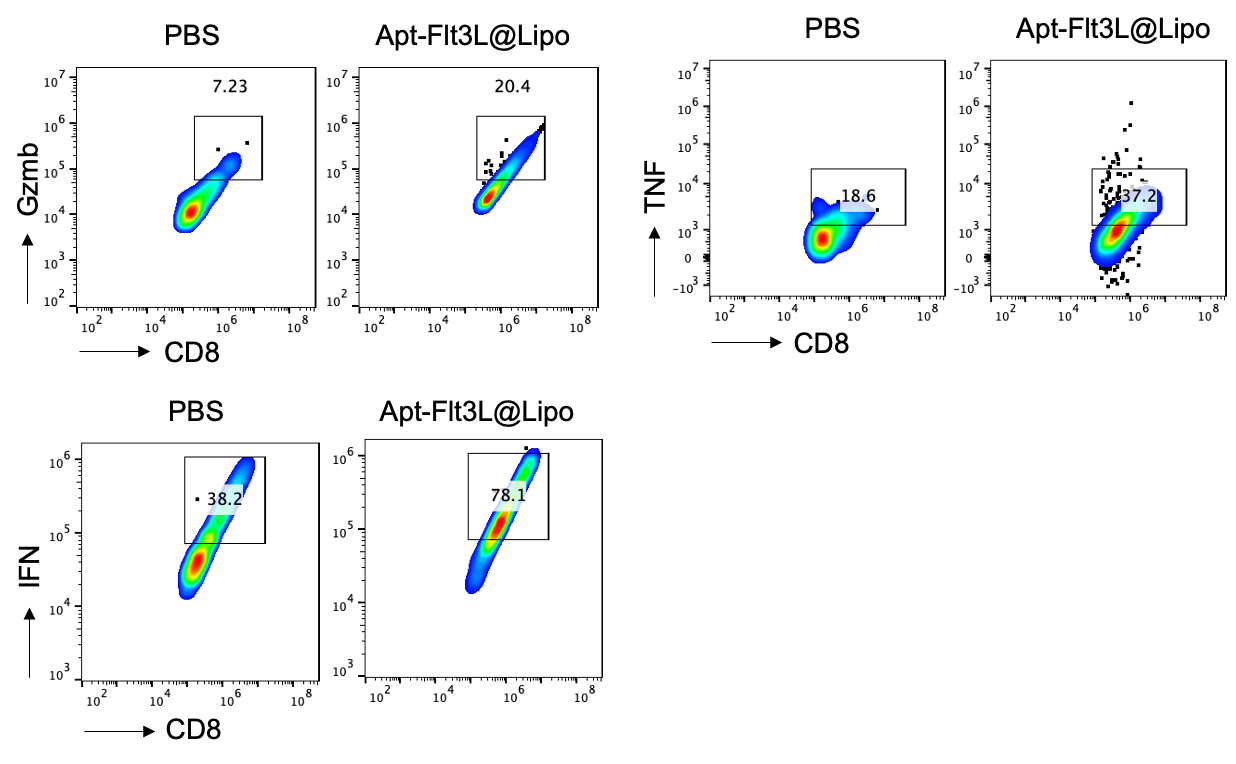
**

**Figure S10.** Flow cytometry analysis of Gzmb^+^ CD8^+^ cells, TNF-α^+^ CD8^+^ cells and IFN-γ^+^ CD8^+^ cells in RM1 tumor treated with Apt-Flt3L@Lipo + US.


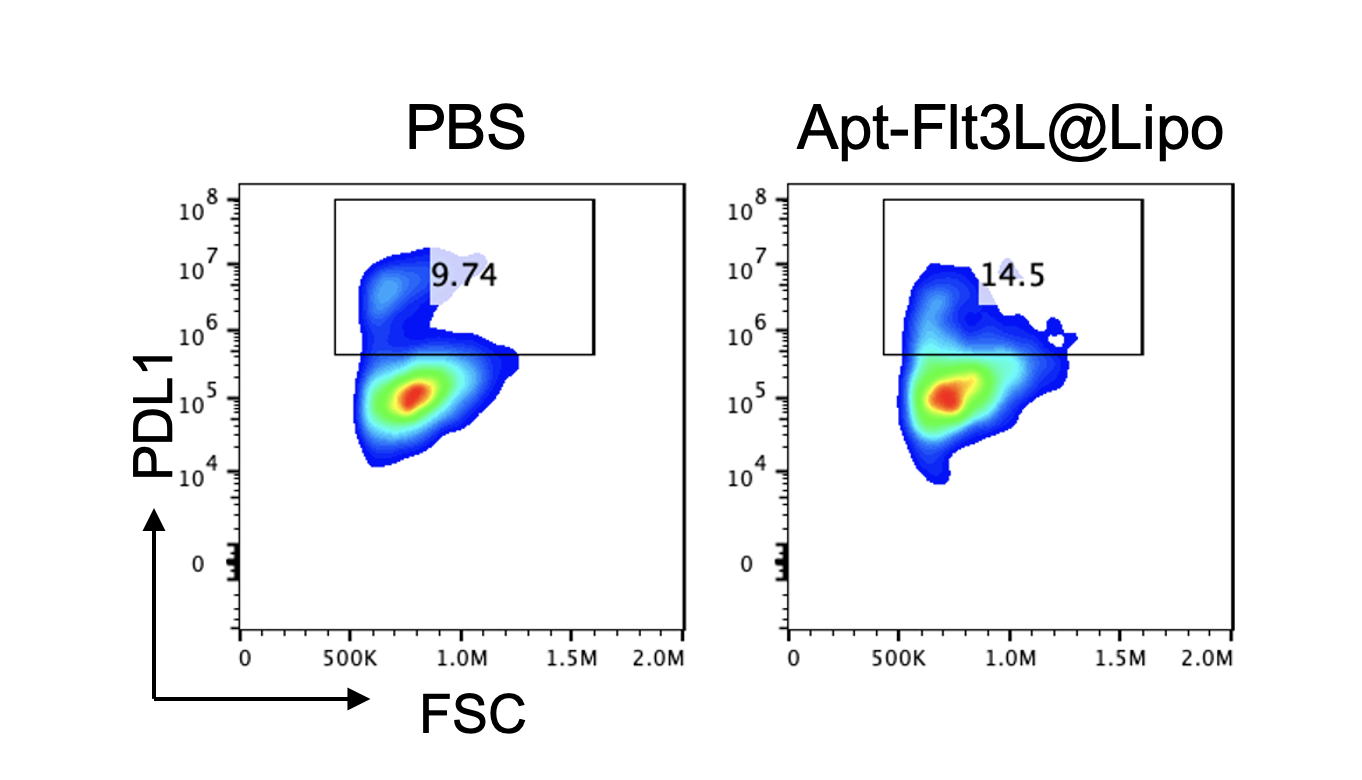


**Figure S11.** After treatment, flow cytometry analysis showed tumor cells in the Apt-Flt3L@Lipo group exhibited significantly higher PD-L1 expression compared to untreated tumors.


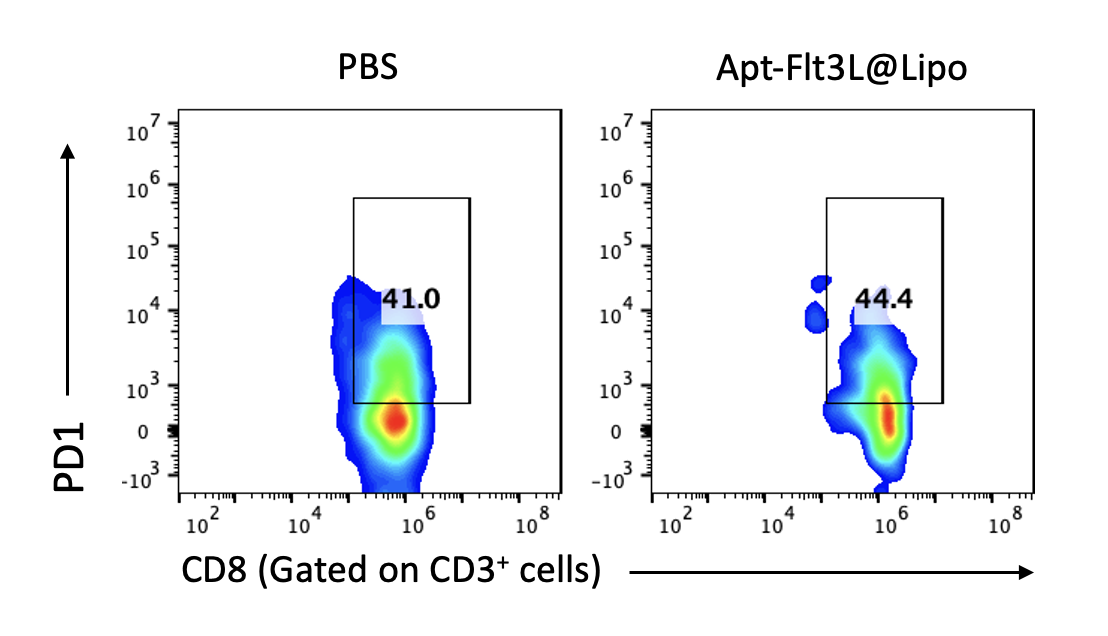


**Figure S12.** After treatment, flow cytometry analysis showed no significant difference of two groups in the expression of PD-1 on T cells in TDLN.

**
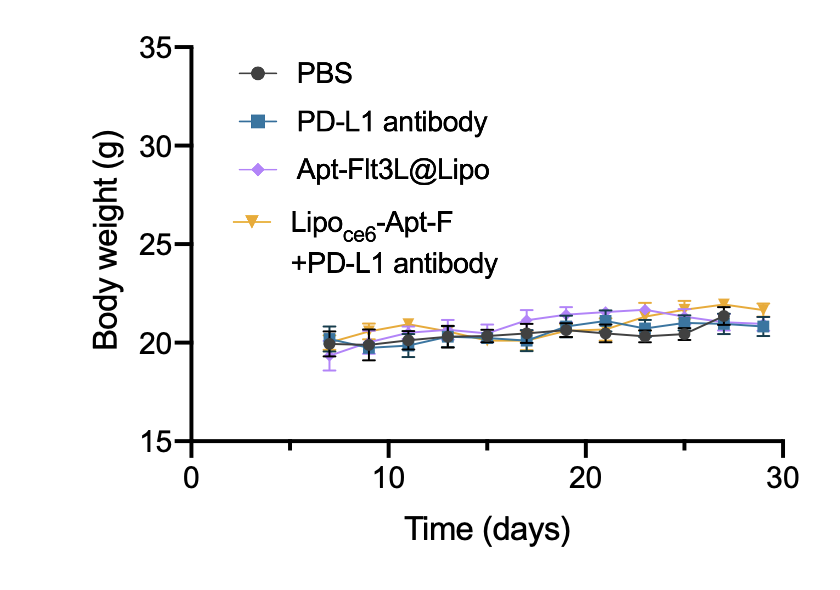
**

**Figure S13.** Mouse body weight across different treatment groups. The treatment strategies of each group are labelled in the figure.

**
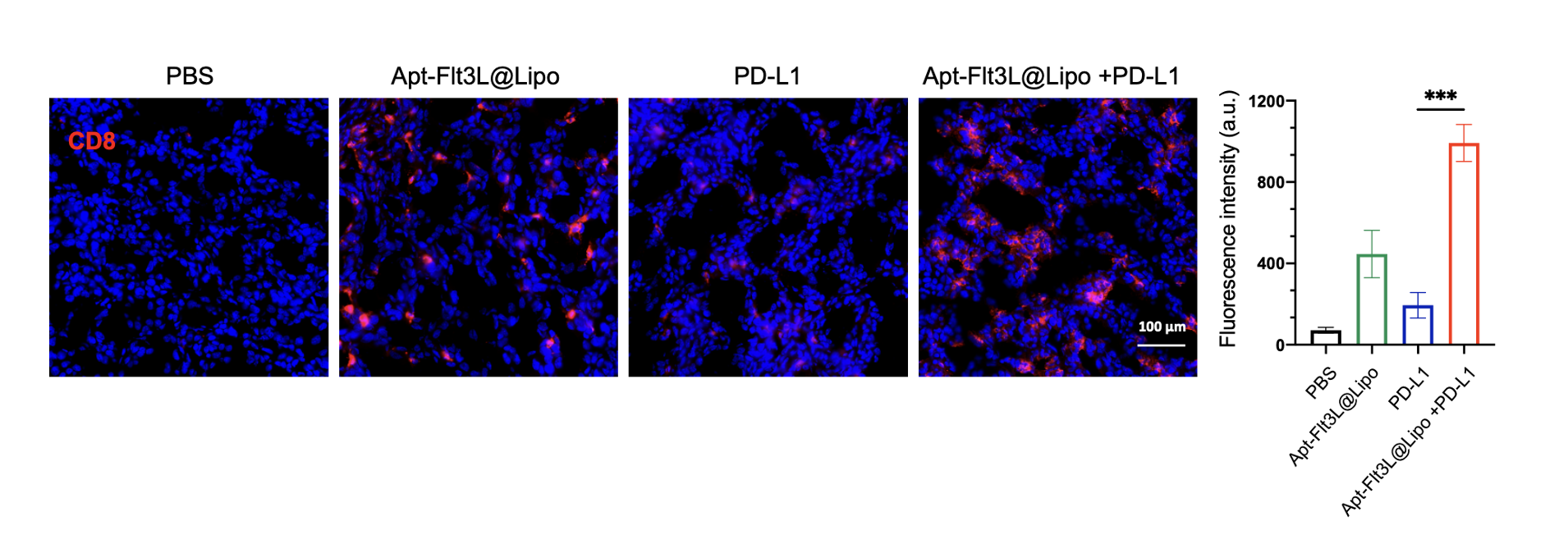
**

**Figure S14.** Representative confocal fluorescence images showing the number of infiltrated CD8^+^ T cells in tumor tissue after different treatments.

**
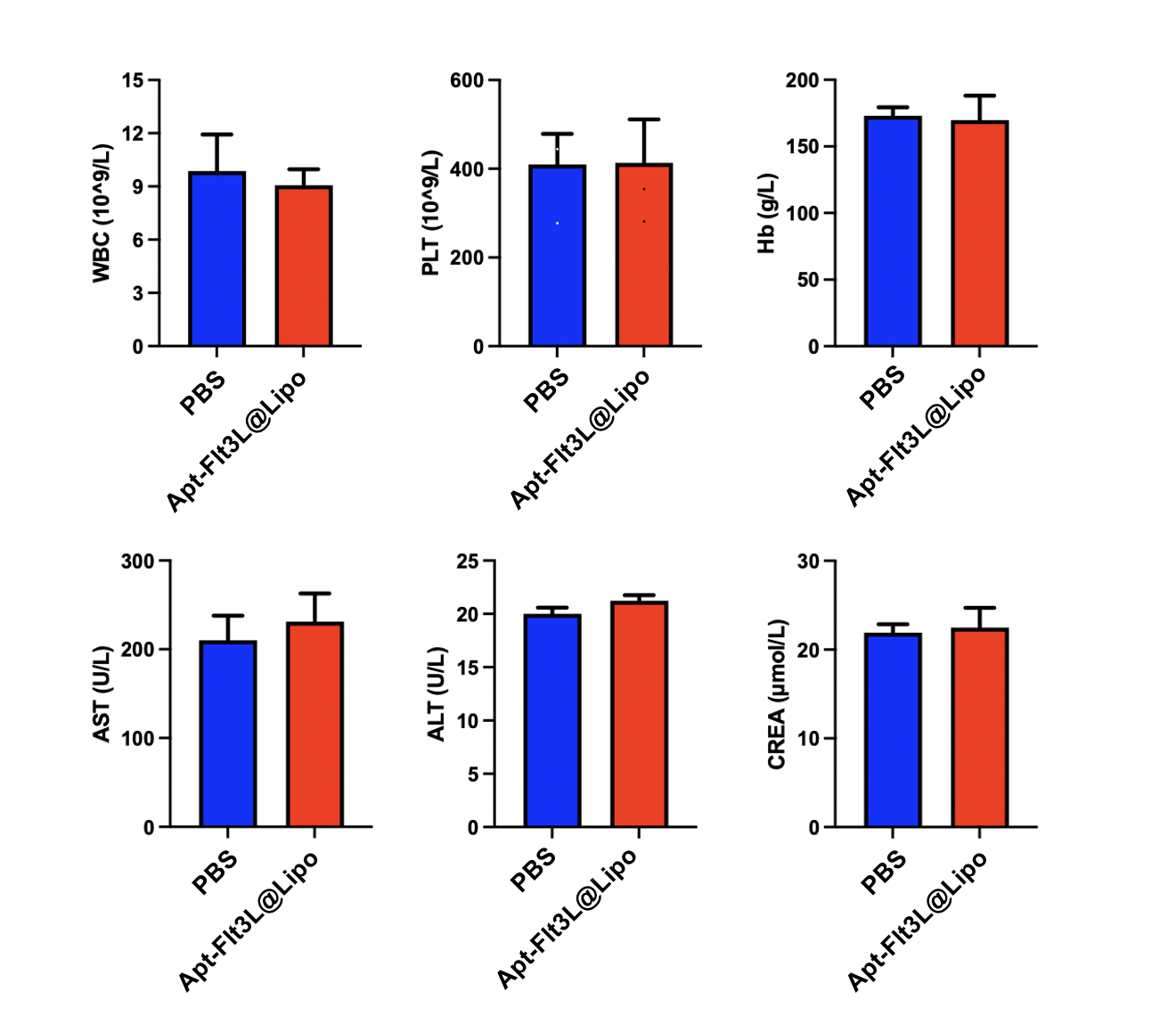
**

**Figure S15.** Serum biochemistry and complete blood panel analysis of the healthy C57 mice after treatment of Apt-Flt3L@Lipo. The liposomes were i.v. injected to the mice. After 24 h, the blood was collected from the mouse orbital for serum biochemistry and complete blood panel analysis.


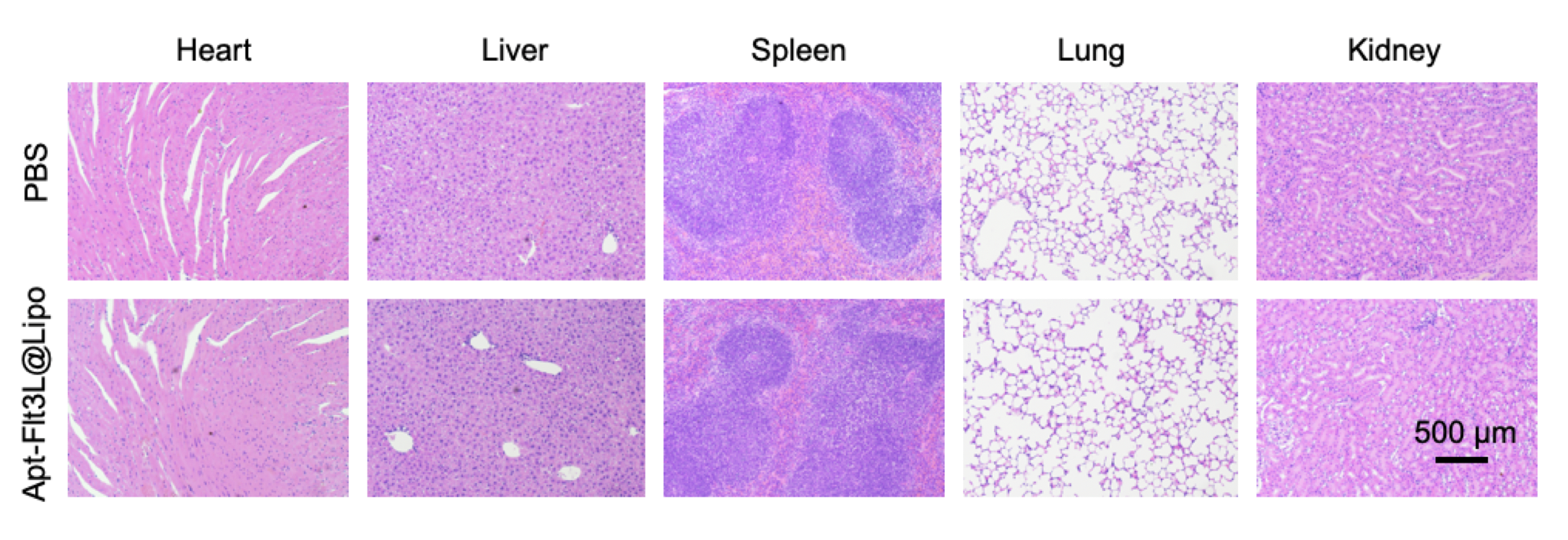


**Figure S16.** Representative hematoxylin and eosin (H&E) staining of major organs, including heart, liver, spleen, lung, and kidney, collected from mice treated with PBS or Apt-Flt3L@Lipo. No obvious pathological abnormalities, tissue damage, or inflammatory infiltration were observed in any of the examined organs.


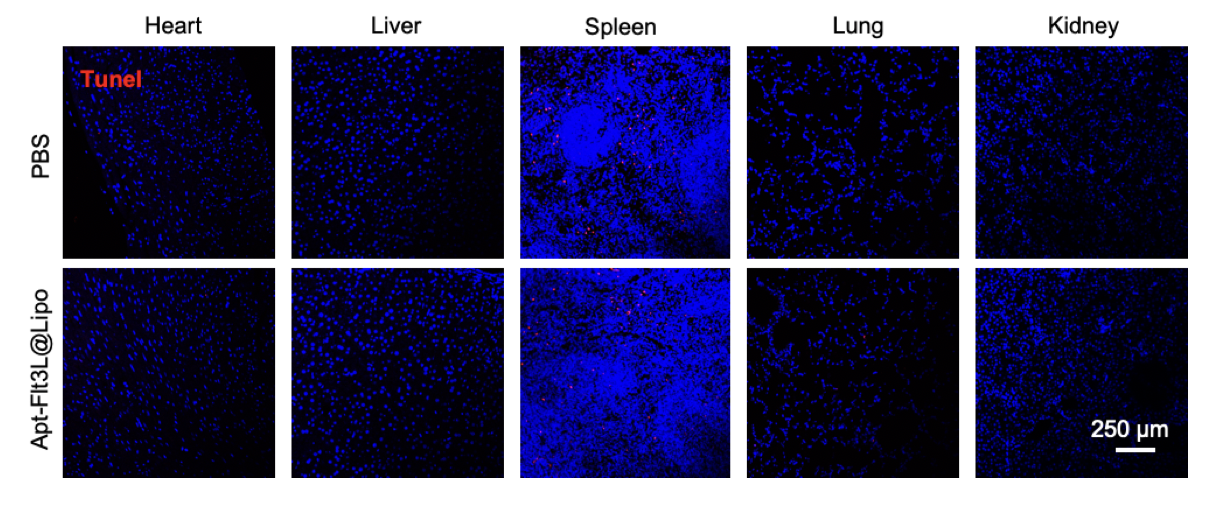


**Figure S17.** Representative TUNEL staining of major organs, including heart, liver, spleen, lung, and kidney, collected from mice treated with PBS or Apt-Flt3L@Lipo, showing negligible apoptotic signals in both PBS- and Apt-Flt3L@Lipo treated groups.

**Figure S18.** Volcano map of differentially expressed genes (DEGs) between the PBS and Apt-Flt3L@Lipo groups (n = 3). The x-axis was the log2 scale of the fold change of gene expression. Negative values indicated downregulation; positive values indicated upregulation. The y-axis was the minus log10 scale of Q values (the adjusted p values), indicating the significant expression difference level. The red dots represented significantly upregulated genes with at least two-fold change, while the blue dots represented significantly downregulated genes with at least two-fold change.


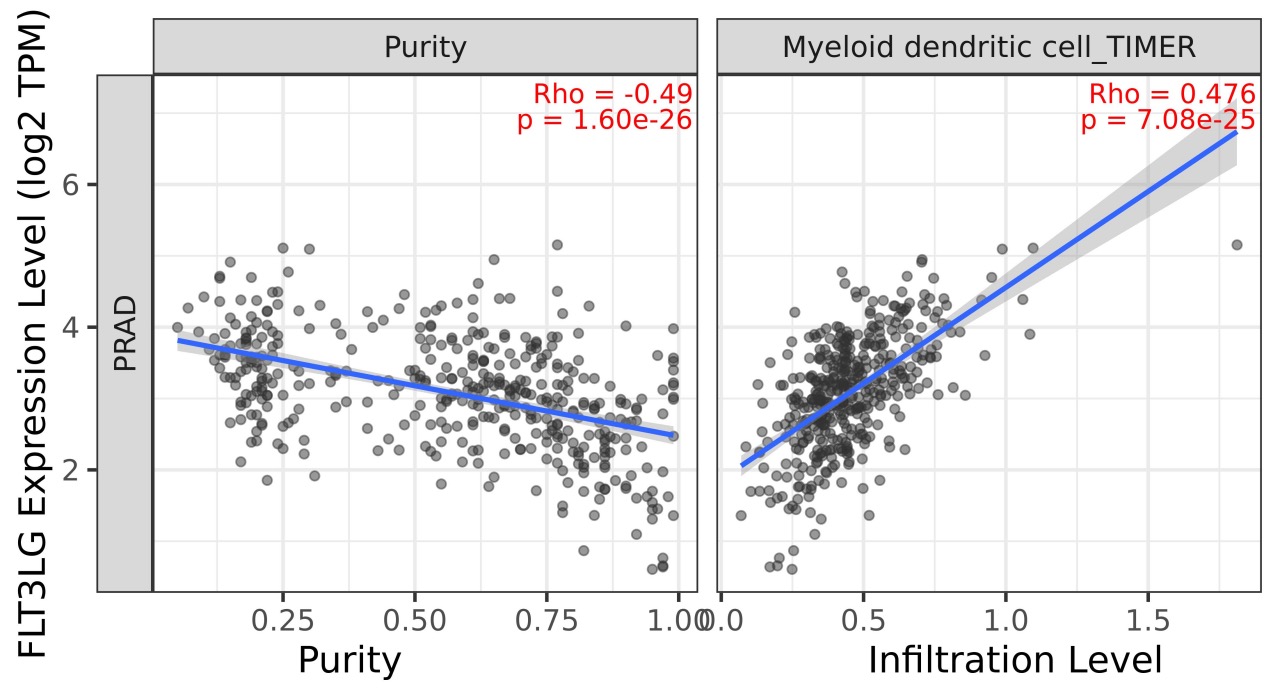


**Figure S19.** Analysis of data from the TCGA database revealed correlation between FLT3LG expression and tumor purity (proportion of tumor cells), and myeloid dendritic cell (mDC) infiltration.


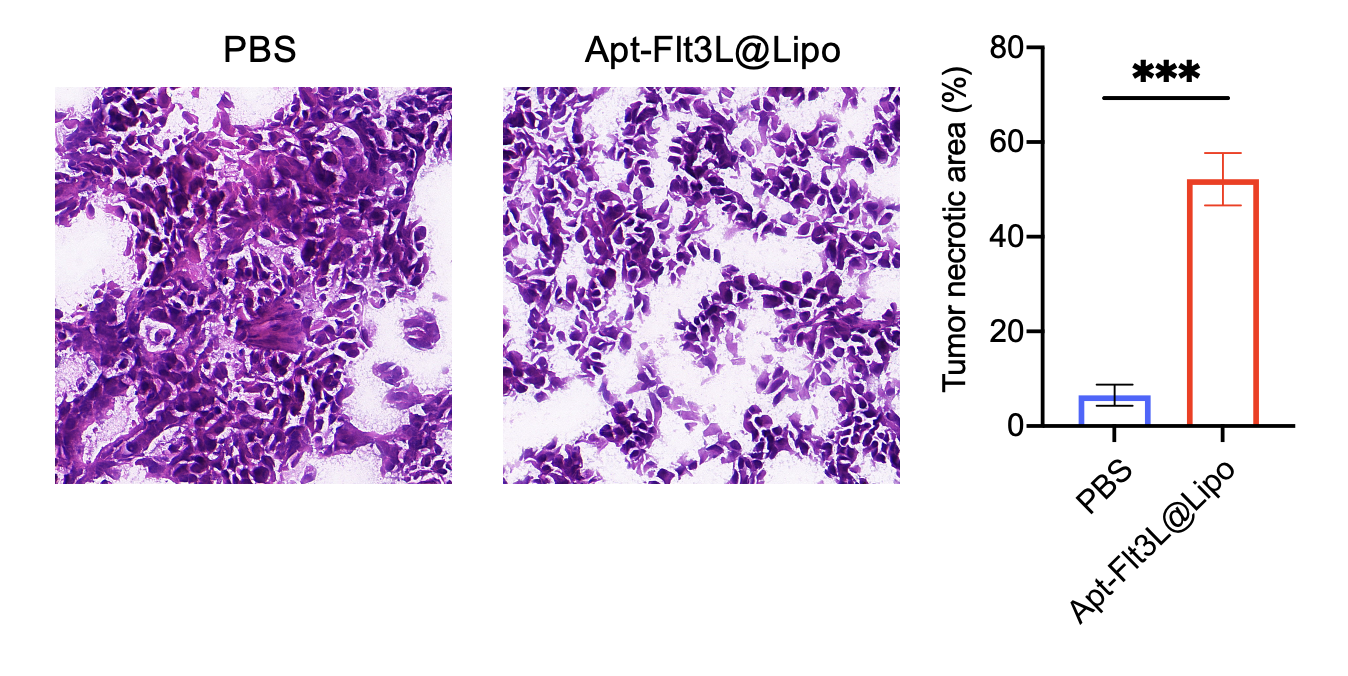


**Figure S20.** H&E staining showed Apt-Flt3L@Lipo disrupted the original morphology of tumor cells and increased necrotic regions within the tumor.


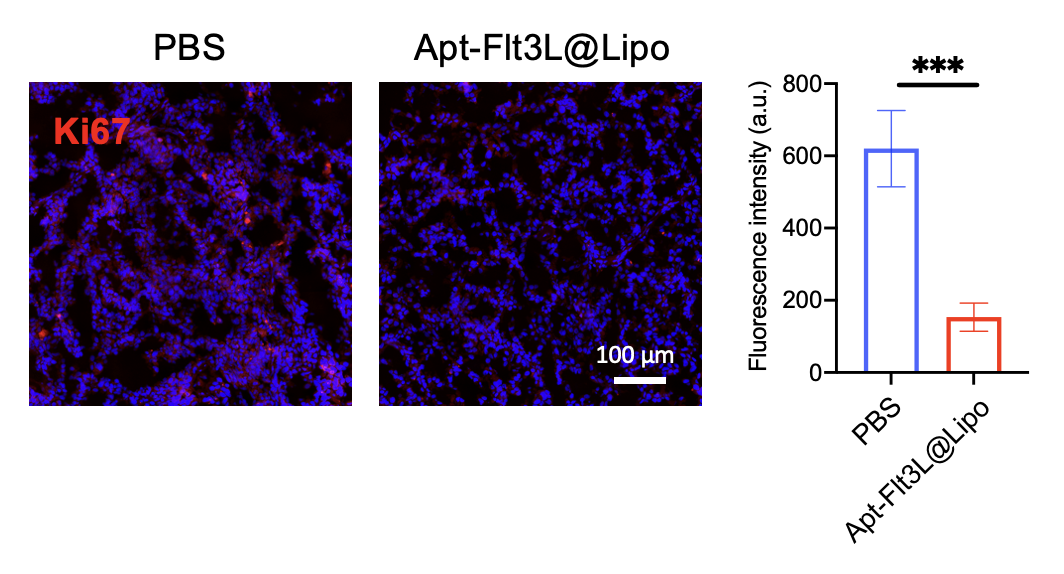


**Figure S21.** Immunofluorescence staining for Ki67^+^ cells (red) in tumor in PBS and Apt-Flt3L@Lipo group and quantification analysis.
